# Ticks and the city - are there any differences between city parks and natural forests in terms of tick abundance and prevalence of spirochaetes?

**DOI:** 10.1186/s13071-017-2391-2

**Published:** 2017-11-21

**Authors:** Maciej Kowalec, Tomasz Szewczyk, Renata Welc-Falęciak, Edward Siński, Grzegorz Karbowiak, Anna Bajer

**Affiliations:** 1 Department of Parasitology, Institute of Zoology, Faculty of Biology, University of Warsaw, 1 Miecznikowa Street, 02-096 Warsaw, Poland; 2 W. Stefański Institute of Parasitology of the Polish Academy of Sciences, 51/55 Twarda Street, 00-818 Warsaw, Poland

**Keywords:** *Ixodes ricinus*, *Borreliella*, *Borrelia miyamotoi*, City, Urban, Natural, Borreliosis, Risk factors

## Abstract

**Background:**

*Ixodes ricinus* ticks are commonly encountered in either natural or urban areas, contributing to Lyme disease agents *Borreliella* [(*Borrelia burgdorferi* (*sensu lato*)] spp. and *Borrelia miyamotoi* enzootic cycles in cities. It is an actual problem whether urbanization affects pathogen circulation and therefore risk of infection. The aim of the study was to evaluate main tick-borne disease risk factors in natural, endemic areas of north-east (NE) Poland (Białowieża) and urban areas of central Poland (Warsaw), measuring tick abundance/density, prevalence of infection with spirochaetes and diversity of these pathogens in spring-early summer and late summer-autumn periods between 2012 and 2015.

**Methods:**

Questing *I. ricinus* ticks were collected from three urban sites in Warsaw, central Poland and three natural sites in Białowieża, NE Poland. A total of 2993 ticks were analyzed for the presence of *Borreliella* spp. and/or *Borrelia miyamotoi* DNA by PCR. Tick abundance was analyzed by General Linear Models (GLM). Prevalence and distribution of spirochaetes was analyzed by Maximum Likelihood techniques based on log-linear analysis of contingency tables (HILOGLINEAR). Species typing and molecular phylogenetic analysis based on the sequenced *flaB* marker were carried out.

**Results:**

Overall 4617 *I. ricinus* ticks were collected (2258 nymphs and 2359 adults). We report well established population of ticks in urban areas (10.1 ± 0.9 ticks/100 m^2^), as in endemic natural areas with higher mean tick abundance (16.5 ± 1.5 ticks/100 m^2^). Tick densities were the highest in spring-early summer in both types of areas. We observed no effect of the type of area on *Borreliella* spp. and *B. miyamotoi* presence in ticks, resulting in similar prevalence of spirochaetes in urban and natural areas [10.9% (95% CI: 9.7–12.2%) *vs* 12.4% (95% CI: 10.1–15.1%), respectively]. Prevalence of spirochaetes was significantly higher in the summer-autumn period than in the spring-early summer [15.0% (95% CI: 12.8–17.5%) *vs* 10.4% (95% CI: 9.2–11.6%), respectively]. We have detected six species of bacteria present in both types of areas, with different frequencies: dominance of *B. afzelii* (69.3%) in urban and *B. garinii* (48.1%) in natural areas. Although we observed higher tick densities in forests than in maintained parks, the prevalence of spirochaetes was significantly higher in the latter [9.8% (95% CI: 8.6–11.0%) *vs* 17.5% (95% CI: 14.4–20.5%)].

**Conclusions:**

Surprisingly, a similar risk of infection with *Borreliella* spp. and/or *B. miyamotoi* was discovered in highly- and low-transformed areas. We suggest that the awareness of presence of these disease agents in cities should be raised.

**Electronic supplementary material:**

The online version of this article (10.1186/s13071-017-2391-2) contains supplementary material, which is available to authorized users.

## Background

*Borrelia burgdorferi* (*sensu lato*) spirochaetes are a complex of Lyme disease (LD) causative agents transmitted by ticks. Among the 21 species of these spirochaetes registered worldwide [1–4], three are responsible for almost all cases of human borreliosis (LD) in Europe: *B. burgdorferi* (*sensu stricto*), *B. afzelii* and *B. garinii* [5]. Recently, the genus *Borrelia* was divided into two genera: *Borrelia*, comprising all relapsing fever (RF) spirochaetes and new genus *Borreliella* [2, 6] including all species of the *B. burgdorferi* (*sensu lato*) complex. The division was supported by the wide molecular analyses of either selected molecular markers or whole genomes, as well as on the basis of ecological features of species and their pathogenicity [2, 7]. Because the type species for *Borrelia* is *B. anserina*, belonging to the RF group, LD-spirochaetes were excluded from the genus *Borrelia* and obtained a new name which replaces the term ‘*B. burgdorferi* (*s.l.*)’. For these reasons, in present paper we will use *Borreliella* for LD-causative bacteria and *Borrelia* when referring to RF-causative agents, including *Borrelia miyamotoi*.

In Poland, as in the whole of central and western Europe, *Ixodes ricinus* ticks constitute the main vector of LD-spirochaetes. Eight *Borreliella* species were detected to date in *I. ricinus* ticks and vertebrate hosts in Poland: *B. afzelii*, *B. bavariensis*, *B. burgdorferi*, *B. garinii*, *B. lusitaniae*, *B. spielmani*, *B valaisiana* and *B. turdii* [8, 9]. These species exhibit different pathogenicity and host specificity, e.g. *B. lusitaniae* is commonly found in lizards, *B. garinii* and *B. turdii* are associated with birds, while *B. afzelii* and *B. burgdorferi* are detected mainly in rodents [10]. However, there are a limited number of studies on the particular species prevalence in Poland, providing information only on *B. burgdorferi* (*s.l.*) complex, current genus *Borreliella*. Nevertheless, during last 15 years the incidence of LD in Poland has risen from 1850 cases in the year 2000, to 4407 in 2005 and 9011 in 2010, to almost 14,000 cases in 2014. In 2016 the number of cases reached 21,000 [11]. Besides LD-spirochaetes, *Ixodes* ticks in the whole of the northern hemisphere transmit the RF-causative agent *B. miyamotoi.* It was isolated for the first time from *I. persulcatus* ticks in Japan in 1995 [12], later also found in ticks in Europe [13, 14], and recently has been recognized as a human pathogen. First, 46 cases of *B. miyamotoi* infection in humans were described in Russia in 2011 [15]. Two years later, *B. miyamotoi* was found in 50 patients with symptoms of relapsing fever with high temperature in the USA, Netherlands and Japan [16–18]. Manifestation of *B. miyamotoi* infection is similar to human granulocytic anaplasmosis (HGA) or tick-borne encephalitis, and was recently referred to as ‘*B. miyamotoi disease*’ (BMD) [19, 20].

Co-occurrence of *Borreliella* spp. and *B. miyamotoi* in *I. ricinus* ticks may also have affected the previously conducted studies on prevalence of LD-causative agents in ticks, particularly the results published before wide recognition of *B. miyamotoi* in ticks in Europe in 2002 [13], as *B. miyamotoi* were not differentiated from other borreliae (*Borreliella* spp.). Molecular resemblance of *B. miyamotoi* and *Borreliella* spp. may also have caused misinterpretation of the results of sero-prevalence studies in tick-bitten persons [21]. It is plausible that the same mechanism was responsible for quite late recognition of *B. miyamotoi* infection in patients with clinical symptoms of a disease after a tick bite [19, 20].

Importantly, *Ixodes* ticks presence is commonly reported in urbanized areas such as suburban forests and city parks [22–30]. While progressive environmental changes and urbanization process increase human exposure to ticks, we do not know how these affect tick-borne pathogens circulation and transmission [29]. Fragmentation of forests is discussed as a factor limiting biodiversity and therefore tick abundance; however no effect of fragmentation on prevalence of LD-spirochaetes was observed in recent study [31]. Despite relatively low biodiversity of ticks and mammals in urban areas, it is possible that LD risk in these habitats is not much different than that in natural areas and could be quite high in cities [27] and within urban space which is not commonly associated with tick-borne disease risk [22].

The risk of acquiring tick-borne disease depends on pathogen, reservoir and vector presence in the environment. All three factors may be affected by urbanization, other environmental transformations and direct human presence [32]. Therefore, it is important to monitor these risk factors in both natural and urbanized areas. Investigation on whether *B. miyamotoi* is present in ticks in frequently visited foci is equally important as study on detection of LD-agents, both actions aiming at focusing attention of physicians and diagnosticians on new possible disease/pathogen diagnosis, in concordance with contemporary conception of ‘One Health’ [33, 34].

The aim of our study was to assess and compare risk factors, i.e. tick abundance and prevalence of infection with *Borreliella* spp. and/or *B. miyamotoi* spirochaetes, in natural areas of north-east (NE) Poland and an agglomeration area in central Poland. An additional aim of our study was to evaluate the diversity of spirochaetes in these two ecologically different types of areas.

## Methods

### Field study

#### Tick collection and research areas

**Fig. 1:**
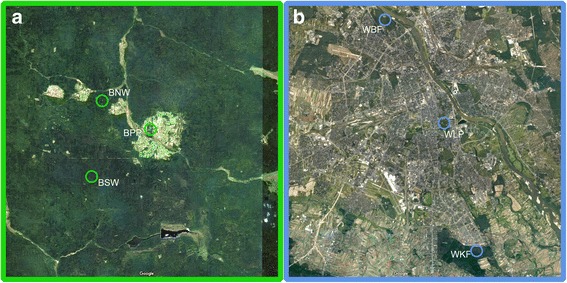
Environment matrix of the study sites. **a** Natural areas of NE Poland (Białowieża and surrounding forests). **b** Urban areas of Central Poland (Warsaw agglomeration). Satellite images are given at the same scale. Map data: Google Maps. *Abbreviations*: BNW, Białowieża North-West; BPP, Białowieża Palace Park; BSW, Białowieża South-West; WBF, Warsaw Bielański Forest; WKF, Warsaw, Kabacki Forest; WLP, Warsaw Łazienki Park

*Ixodes ricinus* ticks were collected in 4-year period, between 2012 and 2015, by flagging in selected semi-natural areas of NE Poland and urban areas of central Poland. Six sites were monitored, three in urban forests or city park in Warsaw and three in forests and city park in Białowieża area. Flagging was performed on surfaces of 50–600 m^2^ with a 1 m^2^ flag in two tick-activity periods: spring-early summer and late summer-autumn. The first season of tick activity, spring-early summer peak, involved collections from March 21st (earliest sampling) to July 31st, the second comprised period between August 1st and October 31st (latest sampling). The length of the whole sampling period reflects the length of vegetation period in Poland. Designated sampling seasons take into account tick summer diapause (hot and dry continental summer in Poland) followed by changes in vegetation structure. Ticks were not collected during and shortly after rainfall. Ticks were identified to species and stage level [35, 36], counted, and tick densities were calculated per 100 m^2^ for each individual flagging event (each visit at specific site). Two types of areas were compared in the study: urban forests/park in Warsaw agglomeration (central Poland) and semi-natural forest/park areas near Primeval Białowieża Forest in NE Poland. Selected urban and natural sites differed, particularly in level of human impact as expressed by the level of human-derived landscape transformation. The matrix [37] of semi-natural areas involved natural and managed forests and low-transformed settlement foci (Fig. 1a). The matrix of urban areas involved highly transformed areas of urban infrastructure, residential areas, streets or arable land (Fig. 1b). In each type of area, 3 study sites were selected, two forest sites (Subtype 1: forest) and one park (Subtype 2: park), representing a gradient of human impact among each area: from undisturbed or moderate (forests) to relatively high (parks).

The main difference (beside localization in urban or natural areas) noted between urban and semi-natural sites could be expressed by everyday activity of humans at each site. All three urban sites are characterized by high numbers of people performing different activities in the forests/parks, especially walking dogs several times a day, walking with children, cycling, etc. Among urban sites, only Kabacki Forest is large enough to avoid human presence in every part of the forest, and in this case human activities may be particularly increased during the weekend period. On the contrary, forest sites around Białowieża town have much lower level of every-day-activity of humans, and are visited mainly by forestry workers, tourists or mushroom pickers, during selected periods of the year.

#### Natural and semi-natural areas near Białowieża town

Białowieża Forest (Białowieża National Park; BNP) (52°46′20″N, 23°50′60″E) (10,517.27 ha) is a residual primeval forest. In 1972 it was added to UNESCO World Heritage List. This region of NE Poland is considered as borreliosis endemic area. Ticks were collected at 3 selected sites distant from each other (Fig. 1a).

The first site, Białowieża Palace Park (BPP) (52°42′24.6″N, 23°50′42.6″E), is a fenced, maintained, regularly mowed park in Białowieża town. The park history dates to the eighteenth century. It was founded by a Russian tsar. Now it is frequently visited by tourists as one of local touristic attractions. It constitutes the island of transformed, wooded environment in this matrix. However, the park is situated in a close vicinity of forest nature reserve (BNP).

The second forest site, Białowieża, North-West (BNW) (52°43′41.0″N, 23°47′19.0″E) is comprising a forest ecotone adjacent to recreational area, localized north-west from Białowieża, mowed partially from the east side and occasionally visited by tourists or forestry workers.

The third natural site, Białowieża, South-West (BSW) (52°39′40.1″N, 23°46′06.2″E) is a forest path inside protected forest area, rarely visited by humans, though distant from nature reserve.

BNP and surrounding forests are known for being inhabited by numerous large mammals, particularly a free-living population of the European bison (*Bison bonasus*), as well as roe (*Capreolus capreolus*) and red deer (*Cervus elaphus*) or wild boar (*Sus scrofa*). Wolves (*Canis lupus lupus*), elks (*Alces alces*) and lynxes (*Lynx rufus*) are permanent inhabitants of the forest, which is also the habitat of many rodents, insectivores, numerous bird species and reptiles [38].

#### Urban, highly transformed areas

Central Poland, Warsaw capital city. Urban and highly transformed areas constitute the matrix of urban sites in the study (Fig. 1b). All selected urban forest habitats are inhabited by rodent and avian hosts, as well as synanthropic carnivores such as red foxes (*Vulpes vulpes*), martens (*Martes foina*) and hedgehogs (*Erinaceus europaeus*). Larger mammals such as roe deer may also occur.

The first urban site, Bielański Forest (WBF) (52°17′32″N, 20°57′36″E), is a small urban forest (152 ha) in north districts of Warsaw, close to Vistula River (Fig. 1b). It comprises the paths of primeval deciduous forest and is under law protection as a nature reserve. Two medium size academic centers (Cardinal Stefan Wyszyński University and Józef Piłsudski University of Physical Education) are placed within the area of WBF. WBF is a place of recreation and physical activities of capital residents. The forest is rich with small mammal species like red squirrels (*Sciurus vulgaris*), *Apodemus* mice (*A. agrarius* and *A. flavicollis*). Large mammals like elk and roe deer were recorded, probably due to proximity of Kampinoski National Park in the North-West.

The second urban site, Kabacki Forest (WKF) (52°6′58″N, 21°3′26″E), is a relatively large managed forest (903 ha) at the south border of the Polish capital city (Fig. 1b). It is a place of recreation and physical activities of residents of the adjacent highly populated residential areas (Ursynów, Kabaty quarters). It is under protection as a landscape reserve. Besides common hosts, it is dwelled by wild boars, roe deer and lizards.

The third urban site, Royal Łazienki Park (WLP) (52°12′53″N, 21°01′58″E), is a vast city park (76 ha), placed near the centre of the capital city (Fig. 1b), the second most frequently visited park in Poland; the attendance of visitors in the palace-park complex is estimated at over 2 million people per year [39]. WLP is carefully managed by a municipality, characterized also by open mowed areas. Classical music concerts are a regular part of summer activities in the park. The park is fenced, protected by park guards and no dogs are allowed inside. It is populated by potential tick hosts such as striped field mouse (*A. agrarius*), red squirrels, hedgehogs, dozen bird species [40]. Even the presence of roe deer has been reported.

### Laboratory study

A representative number of collected ticks (65%) were subjected to the molecular study. Genomic DNA from ticks was isolated with Genomic Tissue Spin-Up kit (AA Biotechnology, Gdynia, Poland) according to the manufacturer’s protocol, from individual adults and from pools of 10 nymphs. Genomic DNA was used for molecular screening for spirochaetes by amplification of pathogen *16S* rDNA with published primers [41], but in modified reaction conditions as follows: initial denaturation in 95 °C for 5 min, 40 cycles of denaturation in 95 °C for 30 s, 30 s of primers annealing in 53 °C and elongation in 72 °C for 30 s. Subsequently, positive samples were analyzed by nested PCR with the use of *Borreliella* spp. and *B. miyamotoi* flagellin gene (*flaB*) marker, with published primers [42]. Initial PCR conditions were modified as follows: initial denaturation in 95 °C for 5 min, 35 cycles of denaturation in 95 °C for 30 s, 30 s of primers annealing in 52 °C and elongation in 72 °C for 80 s, with final elongation in 72 °C for 7 min. Nested PCR was performed with minor modification: denaturation in 95 °C for 20 s and annealing in 55 °C for 20 s, elongation in 72 °C for 60 s. PCR products were visualized on 1.5% agarose gels stained with Midori Green Stain (Nippon Genetics Europe, Düren, Germany). Primers used in both PCR protocols amplify DNA of both *Borreliella* spp. and *B. miyamotoi*. A representative number of positive samples was subsequently sequenced. Additionally, a gene fragment of outer surface protein A (*ospA*) was amplified and sequenced for confirmation of genotype, with primers and PCR conditions already described [43].

For *B. miyamotoi* detection among positive samples, specific primers for *flaB* marker were designed for the nested reaction: forward BmF (5′-AAC TTG CTG TTC AGT CTG GT-3′) and reverse BmR (5′-TTA ACT CCA CCT TGA ACT GG-3′) (424 bp product). Nested PCR conditions remained unmodified.

### *In silico* analysis

Statistical analysis was performed using IBM SPSS Statistics v. 20.0 software. Differences in tick densities (arithmetic means) were evaluated by ANOVA using models with normal errors. General Linear Model (GLM) of One Variable was used to test main effects of *Year* (2012, 2013, 2014 or 2015), *Season* (spring-summer or summer-autumn), *Type of area* (urban or natural), *Subtype of area* (forest or park) and *Site* (Białowieża, natural: BSW, BNW and BPP; Warsaw, urban: WBF, WKF and WLP).

Prevalence of *Borreliella* spp. and/or *B. miyamotoi* infection (percentage of ticks infected) was analyzed by Maximum Likelihood techniques based on log-linear analysis of contingency tables (HILOGLINEAR). For analysis of the prevalence of *Borreliella* spp. and/or *B. miyamotoi* in ticks, we fitted prevalence of bacteria as a binary factor (infected = 1, uninfected = 0) and then *Year* (3 levels: 2013–2015), *Season* (spring-summer or summer-autumn), *Type of area* (urban or semi-natural) or *Subtype of area* (forest or park) or *Site* (1–6; BSW, BNW, BPP, WBF, WKF and WLP). A minimum sufficient model was then obtained, for which the likelihood ratio of *χ* ^2^ was not significant, indicating that the model was sufficient in explaining the data.

Additionally, the distribution of *Borreliella* spp. and *B. miyamotoi* species among positive samples (frequencies or ratio) was compared between *Years*, *Seasons* and *Types* or *Subtypes of areas* or *Sites* by adding *Species* criterion for positive samples in prevalence analysis using the same method (HILOGLINEAR). The *Species* ratio was tested for each main species detected (species-infected = 1, other species infected = 0) or for each detected species (7 levels). For analysis of distribution of species among natural and urban areas, a Jaccard Index of similarity (JI) was calculated as the number of shared variants of each species present in both urban and natural areas, divided by total number of variants of each species.

Minimum Infection Rate (MIR) was calculated for pools of nymphs; if a sample was positive it was assumed that only one tick specimen in the pool was infected. Additionally, NIP value; Nymphal Infection Prevalence (as acknowledged human disease risk-measure [29]) was estimated. NIP was calculated as follows: π = 1-(1-P)^1/*k*^, where π stands for NIP value, *P* is the ratio of number of infected samples (including pools; *n*) to total number of samples in analysis (Q), and *k* is the number of specimens in the pool (Hauckl’s equation as published before, taking into account possibility of more than one specimen being infected in a pool [44]).

*Borreliella* spp. and *B. miyamotoi* sequences obtained were analysed using BLAST-NCBI and MEGA v.6.06 software [45] was used for sequence alignment and further species typing.

Molecular phylogenetic analyses were performed using Maximum Likelihood method of tree-construction. The evolutionary model was chosen with accordance to the data (following implemented model test in MEGA v. 6.06) and bootstrapped over 1000 randomly generated sample trees. Identical sequences obtained in the study were pooled for analysis.

The new nucleotide sequences have been deposited in the GenBank database under the accession numbers MF150046–MF150082 and KT948321–KT948324.

## Results

### Tick abundance (2012-2015)

During four years of study, 4617 *I. ricinus* ticks were collected: 2258 nymphs, 1164 females and 1195 males, in total 296 collection events: 82 in natural and 214 in urban areas. The overall mean abundance (± standard deviation, SD) was 13.2 ± 0.8, 3.5 ± 2.0, 3.8 ± 2.0 and 6.0 ± 0.5/100 m^2^ for total ticks, females, males and nymphs, respectively.

### GLM model for total tick abundance

#### Year × season × type of area

**Fig. 2:**
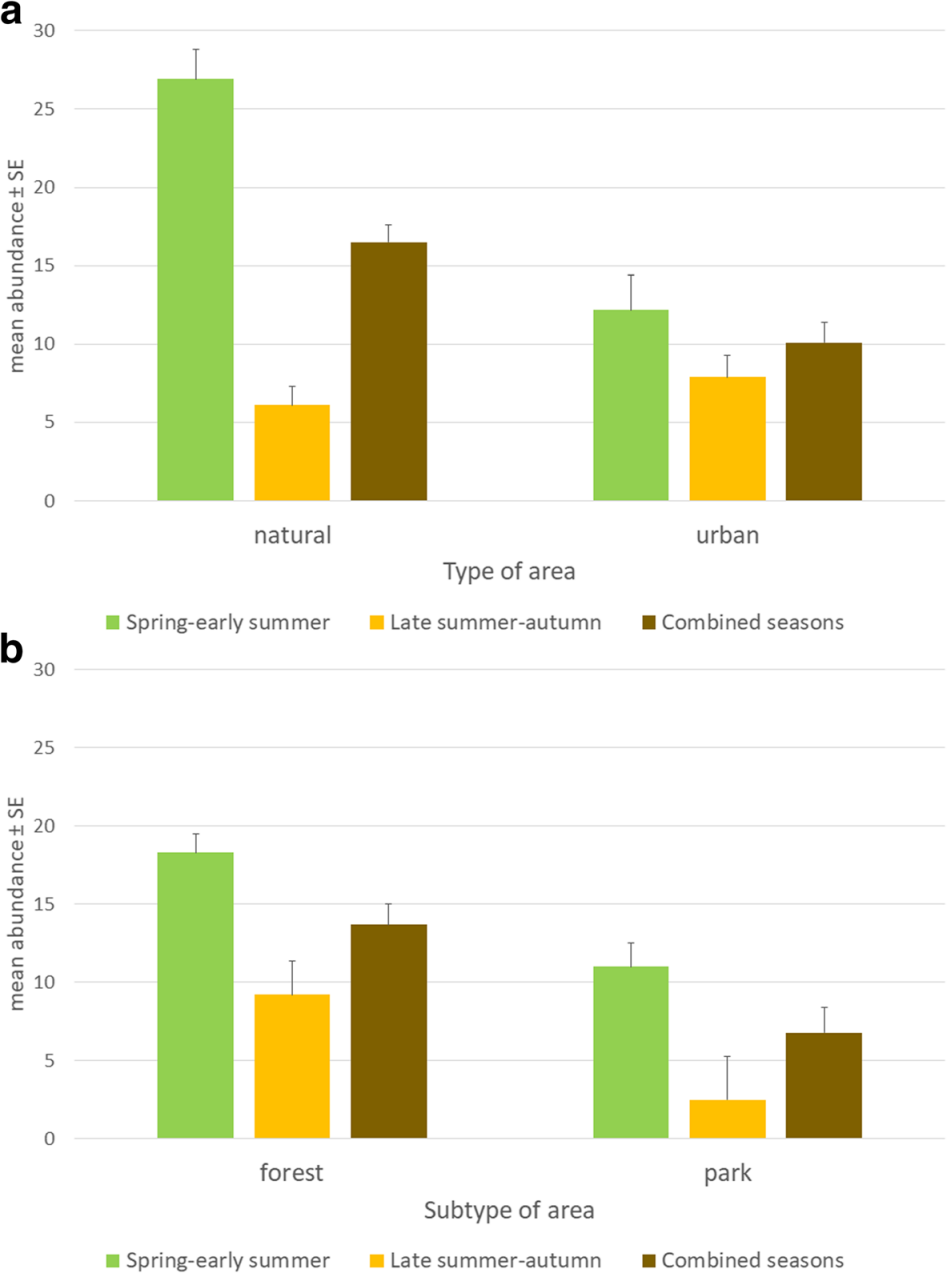
Differences in total tick abundance (no. of ticks/100 m^2^) between *Type* and *Subtype of area* in two seasons. **a** GLM: *Year* × *Season* × *Type of area*. **b** GLM: *Year* × *Season* × *Subtype of area*. GLM statistics provided in Additional file 4: Table S4

Abundance of ticks (nymphs and adults combined) by year and season of the study, and by site and area is presented in Table 1. In tested models, *Year* had an independent strong effect on tick density (main effect of *Year*: *F*_(3,295)_ = 6.9, *P* < 0.001). The highest tick abundance was recorded in 2015, while abundance in the years 2012–2014 was half of 2015 (Table 1). Also, *Season* had a strong effect on tick density (main effect of *Season* on tick density: *F*_(1,295)_ = 57.3, *P* < 0.001). Generally, higher tick abundance was recorded in the first season of tick activity (spring-summer) in comparison to the second one, however it was similar in both seasons in 2013 (Table 1, Fig. 2a).

**Table 1:** Total tick (females, males and nymphs together) abundance (Mean ± SE)

Year	Season^a^	Subtypes^b^/Natural areas				Subtypes^b^/Urban areas				Natural + Urban
		1/Białowieża South-West	1/Białowieża North-West	2/Białowieża Palace Park	Mean Natural	1/Warsaw Bielański Forest	1/Warsaw Kabacki Forest	2/Warsaw Łazienki Park	Mean Urban	Mean
2012	1	47.7 ± 3.3	26.8 ± 4.9	12.6 ± 4.4	33.3 ± 2.8	7.2 ± 3.1	7.8 ± 3.1	nd	7.5 ± 2.7	20.4 ± 2
	2	5.4 ± 5.7	5.0 ± 5.7	0.4 ± 5.7	3.6 ± 4.0	7.7 ± 2.9	1.6 ± 3.3	nd	5.1 ± 2.6	4.4 ± 2.4
	Mean	26.5 ± 3.3	15.9 ± 3.8	6.5 ± 3.6	18.5 ± 2.5	7.5 ± 2.1	4.7 ± 2.3	nd	6.3 ± 1.9	12.4 ± 1.6
2013	1	8.0 ± 4.4	7.4 ± 4.4	2.8 ± 4.4	6.1 ± 3.1	25.5 ± 3.0	12.5 ± 3.1	12.5 ± 3.0	17.0 ± 2.1	11.6 ± 1.9
	2	10.1 ± 7.0	5.1 ± 7.0	3.0 ± 7.0	6.0 ± 4.9	12.2 ± 2.8	9.0 ± 2.9	5.0 ± 2.9	8.8 ± 2.0	7.4 ± 2.7
	Mean	9.1 ± 4.1	6.2 ± 4.1	2.9 ± 4.1	6.1 ± 2.9	18.9 ± 2.1	10.8 ± 2.1	8.7 ± 2.1	12.9 ± 1.5	9.5 ± 1.6
2014	1	33.0 ± 4.9	28.0 ± 4.9	9.3 ± 4.9	23.5 ± 3.5	10.7 ± 3.0	7.0 ± 3.0	10.5 ± 3.1	9.4 ± 2.1	16.4 ± 2.1
	2	7.0 ± 4.9	1.8 ± 4.9	2.0 ± 7.0	3.9 ± 3.8	10.9 ± 3.1	5.7 ± 3.1	2.5 ± 3.5	6.7 ± 2.3	5.3 ± 2.2
	Mean	20.0 ± 3.5	14.9 ± 3.5	5.7 ± 4.3	13.7 ± 2.6	10.8 ± 2.2	6.4 ± 2.2	6.5 ± 2.4	8.0 ± 1.6	10.9 ± 1.5
2015	1	86.1 ± 7.0	40.0 ± 7.0	8.6 ± 7.0	44.9 ± 4.9	20.4 ± 2.8	10.6 ± 2.9	12.8 ± 3.5	15.0 ± 2.1	29.9 ± 2.7
	2	20.0 ± 7.0	10.0 ± 7.0	2.5 ± 7.0	10.9 ± 4.9	17.5 ± 4.9	10.3 ± 4.9	3.1 ± 5.7	11.0 ± 3.6	10.9 ± 3.1
	Mean	53.0 ± 4.9	25.0 ± 4.9	5.5 ± 4.9	27.9 ± 3.5	19.0 ± 2.9	10.5 ± 2.9	7.9 ± 3.4	13.0 ± 2.1	20.4 ± 2
4-year mean	1	43.7 ± 2.6	25.6 ± 2.7	8.3 ± 2.7	26.9 ± 1.9	15.9 ± 1.5	9.5 ± 1.5	12.0 ± 1.9	12.2 ± 1.2	19.6 ± 1.1
	2	10.6 ± 3.1	5.5 ± 3.1	2.0 ± 3.4	6.1 ± 2.2	12.1 ± 1.8	6.7 ± 1.9	3.5 ± 2.5	7.9 ± 1.4	7.0 ± 1.3
	Mean	27.2 ± 2.0	15.5 ± 2.1	5.2 ± 2.2	16.5 ± 1.5	14.0 ± 1.2	8.1 ± 1.2	7.7 ± 1.6	10.1 ± 0.9	13.3 ± 0.9

*Abbreviation: nd* no data

^a^Season codes: 1, spring-early summer; 2, late summer-autumn

^b^Subtype codes: 1, forest; 2, park

GLM statistics is provided in Additional file 4: Table S4

*Type of area* (urban or natural) had an independent effect on the abundance of ticks (main effect of *Type of area* on tick density: *F*_(1,295)_ = 15.2, *P* < 0.001) and was involved in two effect interactions. Mean abundance of ticks was significantly higher in the natural areas near Białowieża, while in city forests and parks of Warsaw the density was about 40% lower (Table 1, Fig. 2a). We have obtained significant effect interaction between *Year* and *Type of area* influencing tick density (*Year* × *Type of area* on tick density: *F*_(3,295)_ = 8.4, *P* < 0.001). Although tick densities were generally higher in natural areas (Fig. 2a), in 2013 similar tick abundance was recorded in both types of areas (Table 1). Another significant effect interaction incorporated three factors (*Year* × *Season* × *Type of area* on tick density: *F*_(3,295)_ = 6.9, *P* < 0.001). Interestingly, although tick abundance was higher in natural habitats in the first season of tick activity, it was very similar both in urban and natural areas in the second season of tick activity (summer-autumn) (Table 1, Fig. 2a).

#### Year × season × subtype of area

*Subtype of area* (forest or park) had an independent effect on the abundance of ticks (main effect of *Subtype of area*: *F*_(1,295)_ = 11.7, *P* = 0.001). Tick abundance in forests was almost 2 times higher than in parks in the study (Fig. 2b, Additional file 1: Table S1). Although abundance in urban and rural parks is similar, it is generally higher in natural forests in comparison to urban forests (Table 1).

#### Year × season × site

There were also significant differences in tick abundance between individual study sites (main effect of *Site* on tick density: *F*_(5,295)_ = 18.9, *P* < 0.001). Both the highest (27.2 ± 2.0 ticks/100 m^2^) and the lowest (5.2 ± 2.2 ticks/100 m^2^) tick abundance was noted among sites near Białowieża town, at the natural forest sites BSW and in BPP, respectively. With the exception of WBF, densities of ticks were generally halved at urban sites, in comparison to natural ones (Table 1). Very similar patterns were observed in the abundance of nymphs and adult ticks. The abundance of nymphs and adults (females and males) is presented in Additional file 2: Table S2 and Additional file 3: Table S3, respectively. Statistical outcomes of GLM analyses are presented in Additional file 4 Table S4.

### Prevalence of spirochaetes (2013-2015)

A total of 4124 *I. ricinus* ticks were collected, of which 2993 specimens (1535 adults and 1458 nymphs) in 1685 samples (860 females, 675 males and 150 pools of nymphs) were screened for spirochaetes with general primers detecting both *Borreliella* spp. and *B. miyamotoi*.

Prevalence of *Borreliella* spp. and/or *B. miyamotoi* in *I. ricinus* ticks (combined, adults, nymphs) in urban and natural sites by *Year* of the study, and by *Site* and *Type/Subtype of area* is presented in Table 2.

**Table 2:** Prevalence of *Borreliella* spp. and *B. miyamotoi* in *I*. *ricinus* ticks in urban and natural sites (2013-2015). HILOGLINEAR statistics provided in Additional file 4: Table S4

Year			2013			2014			2015			Total, all years		
Site	Subtype/Type of area		Total	Adults	Nymphs	Total	Adults	Nymphs	Total	Adults	Nymphs	Total	Adults	Nymphs
Warsaw Bielański Forest	1	Urban	4.2 (26/633)	10.7 (14/132)	2.4 (12/501)	15.1 (48/319)	16.8 (43/257)	8.1 (5/62)	11.5 (20/174)	17.6 (13/74)	7.0 (7/100)	8.4 (94/1126)	15.2 (70/463)	3.7 (24/663)
Warsaw Kabacki Forest	1		6.5 (22/339)	15.9 (20/126)	1.0 (2/213)	15.6 (20/129)	17.9 (18/101)	7.2 (2/28)	15.0 (27/180)	26.3 (21/80)	6.0 (6/100)	10.7 (69/648)	19.3 (59/307)	3.0 (10/341)
Warsaw Łazienki Park	2		17.7 (47/266)	28.8 (46/160)	1.0 (1/106)	13.6 (16/118)	15.4 (15/98)	5.0 (1/20)	20.2 (22/109)	25.4 (21/83)	3.9 (1/26)	17.3 (85/493)	24.1 (82/341)	2.0 (3/152)
Total urban	1 + 2		7.7 (6.2–9.4)	19.1 (14.6–24.7)	1.8 (1.0–3.2)	14.8 (12.6–17.3)	16.7 (12.2–22.2)	7.3 (4.3–12.0)	14.9 (10.7–20.3)	23.2 (19.4–27.4)	6.2 (4.3–8.8)	10.9 (9.7–12.2)	19.0 (16.7–21.3)	3.2 (2.3–4.4)
Total natural	1 + 2	Natural	7.6 (3.9–14.0)	13.4 (7.3–23.0)	3.8 (1.7–7.5)	15.6 (11.8–20.4)	21.1 (17.2–25.5)	1.9 (0.6–5.1)	10.2 (5.7–17.1)	17.2 (9.3–29.1)	3.3 (0.6–11.9)	12.4 (10.1–15.1)	19.1 (14.5–24.7)	3.0 (1.6–5.4)
Białowieża North-West	1		6.8 (4/59)	12.2 (4/33)	0 (0/26)	18.4 (27/147)	22.5 (26/116)	3.3 (1/31)	6.4 (3/47)	8.2 (3/37)	0 (0/10)	13.5 (34/253)	17.8 (33/186)	1.5 (1/67)
Białowieża South-West	1		7.0 (6/86)	14.3 (3/21)	4.7 (3/65)	13.0 (24/186)	19.2 (23/120)	1.6 (1/66)	8.2 (9/110)	18.2 (6/33)	3.9 (3/77)	10.3 (39/382)	18.4 (32/174)	3.4 (7/208)
Białowieża Palace Park	2		11.2 (3/27)	15.4 (2/13)	7.2 (1/14)	18.2 (8/44)	23.6 (8/34)	0 (0/10)	30.0 (6/20)	35.3 (6/17)	0 (0/3)	18.7 (17/91)	25.0 (16/64)	3.8 (1/27)
WBF + WKF + BNW + BSW	1	Forests	5.2 (3.9–6.7)	13.1 (9.9–17.2)	2.1 (1.2–3.6)	15.2 (12.7–18.2)	18.5 (16.0–21.3)	4.8 (2.0–10.8)	11.5 (9.7–13.7)	19.2 (15.8–23.1)	5.6 (3.6–8.4)	9.8 (8.6–11.0)	17.2 (15.0–19.4)	3.3 (2.3–4.4)
WLP + BPP	2	Parks	17.1 (13.4–21.4)	27.7 (20.3–36.5)	1.7 (0.5–5.0)	14.8 (9.4–22.2)	17.4 (12.1–24.3)	3.3 (0.2–17.7)	21.7 (15.9–28.8)	27.0 (18.9–36.5)	3.4 (0.2–16.9)	17.5 (15.1–20.2)	24.2 (19.3–29.9)	2.2 (0.5–7.1)
Total			7.7 (14.7–22.6)	18.4 (13.5–24.3)	2.1 (1.1–3.6)	15.2 (12.3–18.4)	18.1 (15.6–21.4)	4.6 (3.0–6.9)	13.6 (11.4–16.2)	21.6 (17.4–26.4)	5.4 (3.4–8.4)	11.3 (10.2–12.4)	19.0 (17.0–21.0)	3.2 (2.3–4.2)

Prevalence % (no. positive/no. tested; or 95% CI for total urban, total natural, forests, parks and total)

Nymphs: Minimum Infection Rate (MIR) is given

Among five factors implemented into log linear analyses of prevalence in ticks, only *Season* was associated with infection status (*Season* × presence/absence of *Borreliella* spp. and/or *B. miyamotoi*: *χ* ^2^ = 4.3, *df* = 1, *P* = 0.039). A higher percentage of positive ticks was detected in the second season of tick activity (late summer-autumn), 10.4% (95% CI: 9.2–11.6%) *vs* 15.0% (12.8–17.5%).

Interestingly, differences in *Borreliella* spp. and/or *B. miyamotoi* prevalence between years of the study and the two types of areas were not significant (Table 2). Overall prevalence of *Borreliella* spp. and/or *B. miyamotoi* infection was very similar in both urban (11%) and natural areas (12.4%) for all ticks combined (NS). Overall prevalence was almost identical in urban and natural areas, both for adults [about 19% (17.0–21.0%)] and nymphs [MIR about 3% (2.3–4.2%)] (Table 2). Differences in *Borreliella* spp. and/or *B. miyamotoi* prevalence between years in adult ticks were about 5%, differences in MIR in nymphs ranged 2–5% (NS). Additionally, for *Borreliella* spp. and/or *B. miyamotoi* infection in nymphs, identical NIP was calculated both in natural (3.5%; *n* = 9; Q = 31) and urban (3.8%; *n =* 37; Q = 119) areas. The differences between NIPs and MIRs were not significant. Although there were some differences in *Borreliella* spp. and/or *B. miyamotoi* prevalence between individual sites, they were not significant (Table 2). The highest percentage of positive ticks was noted in two parks, WLP (17%) in urban and in BPP (19%) in natural areas, and was reflecting the highest percentage of positive adult ticks (24 and 25%, respectively) (Table 2).

Thus, *Borreliella* spp. and/or *B. miyamotoi* prevalence was significantly higher in parks (subtype 2, more changed habitat) in comparison to forests (subtype 1, less transformed forest) (*Subtype of area* × presence/absence of *Borreliella* spp. and/or *B. miyamotoi*: *χ* ^2^ = 7.6, *df* = 1, *P* = 0.006) (Table 2).

### Species of spirochaetes detected in the study

Species typing was performed on the basis of sequencing of flagellin gene fragment (~600 bp product); 230 of 338 positive PCR samples were sequenced. Alignment and BLAST-NCBI analyses revealed presence of six *Borreliella* species: *B. afzelii*, *B. burgdorferi*, *B. garinii*, *B. lusitaniae*, *B. spielmani* and *B. valaisiana* (Table 3). Additionally, five *B. miyamotoi* sequences were obtained. *Borreliella afzelii* was the dominant species (131/230; 57%) (Table 3), the second most frequent was *B. garinii*, followed by *B. burgdorferi* (Table 3). Other species, like *B. valaisiana*, *B. lusitaniae*, *B. spielmani* and *B. miyamotoi* were relatively rare (< 5%) (Table 3). We grouped these as ‘rare’ in further analysis. *Borrelia miyamotoi* was identified only in samples from WKF (2/648) in urban and BNW (3/186) in natural areas, while *B. spielmani* was found only in one tick from WBF (0.4%).

**Table 3:** Frequency of species in the study. HILOGLINEAR statistics provided in Additional file 4: Table S4

		Lyme disease group (*Borreliella* spp./*B. burgdorferi* *sensu lato*)							Relapsing fever group
Habitat	No. of samples	*B. afzelii*	*B. burgdorferi*	*B. garinii*	*B. lusitaniae*	*B. spielmani*	*B. valaisiana*	Total	*B. miyamotoi*
Natural	77	32.5 (25)	3.9 (3)	48.1 (37)	1.3 (1)	0 (0)	10.4 (8)	96.1 (74)	3.9 (3)
Urban	153	69.3 (106)	17.0 (26)	7.8 (12)	2.0 (3)	0.7 (1)	2.0 (3)	98.7 (151)	1.3 (2)
Natural + Urban	230	57.0 (131)	12.6 (29)	21.3 (49)	1.7 (4)	0.4 (1)	4.8 (11)	97.8 (225)	2.2 (5)
Forest	161	49.7 (80)	13.7 (22)	24.8 (40)	2.5 (4)	0.6 (1)	5.6 (9)	94.4 (152)	3.1 (5)
Park	69	73.9 (51)	10.1 (7)	13.0 (9)	0 (0)	0 (0)	2.9 (2)	97.1 (67)	0 (0)

Frequency of species in % (no. positive samples)

Interestingly, the distribution of species (frequency) differed between natural and urban areas (*Type of area* × species: *χ* ^2^ = 67.6, *df* = 6, *P* < 0.001) (Table 3). *Borreliella afzelii* was present in over 2/3 of positive ticks from urbanized and in almost 1/3 of positive ticks from natural areas (Table 3). Also *B. burgdorferi* was slightly more common in urban/suburban sites than in natural sites (Table 3). However, *B. garinii* was more common in natural sites, being detected in almost half of the positive samples (Table 3). ‘Rare’ species were more frequent at natural sites near Białowieża town (15.6 *vs* 5.8% at urban sites). Three most common *Borreliella* species (*B. afzelii*, *B. burgdorferi* or *B. garinii*) represented the great majority of positive samples in both natural and urban areas: 84.4% (65/77) and 93.5% (143/153), respectively (*χ* ^2^ = 0.3, *df* = 1, *P* = 0.595). Distribution of species differed also between parks and forests (*Subtype of area* × species: *χ* ^2^ = 16.6, *df* = 6, *P* = 0.011). In parks, *B. afzelii* was identified in almost 3/4 of positive ticks, while frequency of *B. burgdorferi* and *B. garinii* was much lower and only few *B. valaisiana* infections were detected. In forests in both types of areas, all seven species of spirochaetes were present, *B. afzelii* was present in a half of samples and *B. garinii* was present in 25% of positive ticks (Table 3).

Among all sequences obtained there were some unclear sequences with ambiguous nucleotides, for which further analysis, in some cases, revealed co-infection of two species: *B. afzelii* and *B. garinii*, as well as *B. valaisiana* and *B. lusitaniae*, *B. burgdorferi* and *B. miyamotoi*. However these additional data were excluded from further phylogenetic and frequency analyses. One case of infection with *B. miyamotoi* was confirmed by sequencing of 424 bp product of flagellin gene fragment with use of *B. miyamotoi*-specific primers (excluded from sequence analysis). There were four more *B. miyamotoi*-positive samples detected, however in most cases sequencing was inconclusive. There were a few discordant results after sequencing the same samples with different primers, e.g. two samples typed by BLAST as *B. miyamotoi* by analysis of 424 bp product of primers specific for *B. miyamotoi*, further typed by BLAST as *B. afzelii* and *B. burgdorferi* in another sample with use of ~600 bp product of general *Borreliella* spp. and *B. miyamotoi* primers. For that reason, positive samples detected by *B. miyamotoi*-specific primers were excluded from distribution or heterogeneity analysis, although overall prevalence of *B. miyamotoi* was estimated 0.33% (10/2993; 95% CI: 0.16–0.61%).

### Sequence analysis and phylogeny

All chromatograms were checked manually for sequence quality. Each sequence containing ambiguous nucleotides was resolved in comparison to the reference sequence of greatest homology (from GenBank BLAST analysis). Following chromatogram reading, the ambiguous nucleotide sites were assigned either in accordance or alternatively to a reference sequence. The alternative sequences were subjected to the secondary BLAST analysis. If the most similar sequence in GenBank was the same species as the reference sequence, the secondary sequence was saved and subjected to further heterogeneity and phylogenetic analyses, while the sample was qualified as multi-strained. If the secondary sequence was most similar to different species, it was excluded from further analyses, while the sample was qualified as co-infected with two species. The 230 samples species-typed by sequence BLAST were involved in analyses of frequency, while the 264 sequences resolved from that 230 samples were used in heterogeneity analysis by Jaccard Index of similarity and were clustered for phylogenetic analysis. Subsequently, a 547 bp consensus alignment was analysed and identical sequences were clustered for further phylogenetic analysis. Overall, 38 unique variants of *flaB* sequence were obtained: five variants of *B. afzelii*, six variants of *B. burgdorferi*, 18 variants of *B. garinii*, one variant of *B. lusitaniae*, five variants of *B. valaisiana*, one variant of *B spielmani* and two variants of *B. miyamotoi* (Table 5). Comparison of the distribution of *flaB* sequences among natural and urban areas revealed that *flaB* sequences of all species, except *B. lusitaniae* (JI = 1), differed between types of areas, although were most similar for *B. afzelii* (JI = 0.6, Table 4).

**Table 4:** Comparison of heterogeneity of *Borreliella* species in the two types of areas studied in Poland (2013–2015)

Species	Sum of variants	Natural	Urban	N + U	Jaccard index
*B. afzelii*	5	4	4	3	0.60
*B. burgdorferi*	6	2	6	2	0.33
*B. garinii*	18	17	6	5	0.28
*B. miyamotoi* ^a^	2	1	1	0	0.00
*B. lusitaniae*	1	1	1	1	1.00
*B. spielmani* ^a^	1	0	1	0	0.00
*B. valaisiana*	5	5	1	1	0.20
Ba + Bb + Bg	29	23	16	10	0.34

*Abbreviation*: *N + U* both in natural and urban

^a^Data not sufficient for heterogeneity comparison

There was minor diversity in *B. afzelii flaB* sequences (544 bp). Among *B. afzelii* sequences (*n* = 143) derived from 131 positive samples, overall five *B. afzelii* variants with similarity levels of 99.4–99.8% (541–543/544 nucleotides) were recognised (Table 5). Our variants were either identical to, or most similar with sequences from Germany, Czech Republic and Poland (Table 5, Additional file 5: Figure S1). Beside two variants, our *B. afzelii* variants were present in samples from both natural and urban areas (Table 5).

**Table 5:** Variants of *flaB* sequences generated in this study

Species	Variant (*flaB*)	*n*	GenBank	Reference in GenBank	Similarity (%)	Nucleotide identity	Type of area	Origin of samples (*n*)
*Ba*	Ba_V1a	118	MF150047	CP018262, KF990318, KX646195, KF894064	100	544/544	NU	BNW (8), BSW (9), BPP (4), WBF (37), WKF (20), WLP (40)
	Ba_V1b	1	MF150048	CP018262, KF990318, KX646195, KF894064	99.8	543/544	N	BSW (1)
	Ba_V2cc	1	MF150049	KR782215, JN828691	99.8	543/544	U	WBF (1)
	Ba_V2ct	9	MF150050	CP018262, KF990318, KX646195, KF894064	99.8	543/544	NU	BNW (1), BSW (2), WBF (2), WLP (4)
	Ba_V2tc	14	MF150051	KR782215, JN828691	100	544/544	NU	BPP (1), WBF (7), WKF (1), WLP (5),
*Bb*	Bb_V1^a^	1	MF150046	CP009656	98.0	536/547 (3 gaps)	U	WKF (1)
	Bb_V2	13	MF150052	KX646201, KF422803, CP001205	100	544/544	NU	BNW (2), WBF (1), WKF (9), WLP (1)
	Bb_V3	5	MF150053	DQ016620, AB035618	100	544/544	U	WBF (1), WKF (2), WLP (2)
	Bb_V5	1	MF150054	CP009656	100	544/544	U	WKF (1)
	Bb_V6	1	MF150055	CP009656	99.8	543/544	U	WKF (1)
	Bb_V7	8	MF150056	DQ016625, KF836508, AB091813, AB052665, CP002312	100	544/544	NU	BNW (1), WKF (3), WLP (4)
*Bg*	Bg_vA	20	MF150057	HM345898, JN828685, AB178327	100	544/544	NU	BNW (8), BSW (5), BPP (2), WBF (2), WKF (3)
	Bg_vAa	3	MF150058	AB091814	100	544/544	NU	BNW (1)
	Bg_vB	11	MF150059	HM345905, KF836512	100	544/544	NU	BNW (3), BSW (4), BPP (3), WKF (1)
	Bg_vBa	1	MF150060	KX646196, KF990320, JN828685, AB178327	99.8	543/544	N	BSW (1)
	Bg_vBb	1	MF150061	HM345905, KF836512	99.8	543/544	N	BPP (1)
	Bg_vC	6	MF150062	KX646196, KF990320, JN828685	99.8	543/544	N	BNW (1), BSW (4), BPP (1)
	Bg_vCa	4	MF150063	KX646196, KF990320, JN828685	99.8	543/544	N	BNW (1), BSW 1 BPP (2)
	Bg_vDa	1	MF150064	KF836510, KF918606	99.8	543/544	N	BSW (1)
	Bg_vDb	1	MF150065	KX646202, JN828682, AB178330	99.3	543/544	N	BSW (1)
	Bg_vDc	1	MF150066	KF990322	100	544/544	N	BSW (1)
	Bg_vDd	4	MF150067	KX646202, JN828682, AB178330, KF894053, KF894052	100	544/544	N	BNW (2), BSW (1), BPP (1)
	Bg_vDe	1	MF150068	KX646202, JN828682, AB178330, KF894053, KF894052	99.4	541/544	N	BSW (1)
	Bg_vDf	2	MF150069	KX646202, JN828682, AB178330, KF894053, KF894052	99.6	542/544	N	BNW (1), BSW (1)
	Bg_vEa	2	MF150070	KF990320, HM345902, JN828685, AB178327, KF894058	99.8	543/544	NU	BSW (2)
	Bg_vEb	3	MF150071	KF894058, HM345901	100	544/544	NU	BNW (1), WBF (1), WKF (1)
*Bg*	Bg_vEc	4	MF150072	KF894061, KF422820, HM345897, AB178326	100	544/544	U	WBF (2), WKF (1), WLP (1)
	Bg_vEd	2	MF150073	KF990320, HM345898, DQ650331, AB178327	99.4	541/544	N	BSW (2)
	Bg_vEf	2	MF150074	DQ650336, DQ016621	99.6	542/544	N	BSW (2)
*Bl*	Bl_V1	4	MF150075	KF422804, DQ016623, HM345914	100	544/544	NU	BSW (1), WKF (3)
*Bm*	Bm_V2^b^	1	KT948324	KX646199, DQ650332	100	538/538	U	WKF (1)
	Bm_V1^b^	4	KT948321-23	KX646199, DQ650332	99.8	537/538	NU	BNW (3), WKF (1)
*Bs*	Bs_V1	1	MF150076	KF422808, JF732881	100	544/544	U	WBF (1)
*Bv*	Bv_V1	7	MF150077	DQ650330, KX646197, AB178333, CP009117	100	544/544	NU	BNW (3), BSW (1), BPP (1), WKF (2)
	Bv_V2	3	MF150078	HM345912, AB091805	99.8	543/544	N	BNW (1), BSW (1), BPP (1)
	Bv_V3	1	MF150079	HM345912, AB091805	100	544/544	N	BSW (1)
	Bv_V4	1	MF150080	KF422808, JF732881	99.8	543/544	N	BSW (1)
	Bv_V6	1	MF150081	KF422808, JF732881	99.6	542/544	N	BNW (1)

*Abbreviations*: *Ba B. afzelii*, *Bb B. burgdorferi*, *Bg B. garinii*, *Bl B. lusitaniae*, *Bm B. miyamotoi*, *Bs B. spielmani*, *Bv B. valaisiana*, *N* natural, *U* urban, *NU* natural and urban, *BNW* Białowieża, North-West, *BSW*, Białowieża, South-West, *BPP* Białowieża, Palace Park, *WBF* Warsaw, Bielański Forest, *WKF* Warsaw, Kabacki Forest, *WLP* Warsaw, Łazienki Park

^a^Longer sequence in the alignment (insertion)

^b^Shorter sequence in the alignment (deletion)

Sequences (*n* = 29) of *B. burgdorferi* (544 or 547 bp) were quite diverse. Six variants were recognized among 29 sequenced samples, the similarity level of variants was 97.6–99.8% (534–543/544 or 547). Our *B. burgdorferi* variants displayed the highest similarity with sequences from the USA, Switzerland, Germany, Poland, Russia or Turkey (Table 5, Fig. 2, Additional file 5: Figure S1). The first variant, Bb_V1 (*n* = 1) displayed highest similarity (only 98% of 547 bp, 3 gaps) with strain B31 from *I. scapularis* from the USA (CP009656). Five of seven of our *B. burgdorferi* variants were reported exclusively in urban sites, mostly in WKF (Table 5). A higher number of *B. burgdorferi flaB* variants was detected in urban areas (Table 4).

*Borreliella garinii* was the most heterogenic species. Eighteen variants of *flaB* sequence (544 bp) were recognized among 69 *B. garinii* sequences obtained from 49 sequenced samples. Our *B. garinii* variants displayed the highest similarity with sequences from Czech Republic, Poland and Russia, as well as from Turkey, mostly from *I. ricinus* ticks and *Apodemus* spp. mice (Table 5, Additional file 5: Figure S1). One variant (Bg_vEc from WBF) was present exclusively in urban areas (Table 5). The other 17 *B. garinii* variants were present in natural areas; five of these were recorded also in urban areas (Table 5). The number of *flaB* variants of *B. garinii* was higher in natural areas (Table 4).

The only variant of *B. lusitaniae* (*n* = 4) was identical with previously obtained sequences from *I. ricinus* from Poland (KF422804, DQ016623, HM345914) and was present in both urban (WKF, *n* = 3) and natural (BSW, *n* = 1) areas, so distribution was identical in both types of areas (Table 4).

The single *B. spielmani* sequence obtained from urban WBF was identical with sequences from *I. ricinus* from France (KF422808) and red fox (*Vulpes vulpes*) from Poland (JF732881).

Among *B. valaisiana* sequences (*n* = 12) from 11 positive samples, five variants were identified (Table 5). Our *B. valaisiana* sequences displayed the highest similarity with sequences from Poland, Russia and Turkey (Table 5, Additional file 5: Figure S1). Variant Bv_V1 was present either in urban or natural areas. All other variants were detected exclusively in natural areas. Thus, a higher number of *B. valaisiana flaB* variants was detected in natural areas (Table 4).

**Fig. 3:**
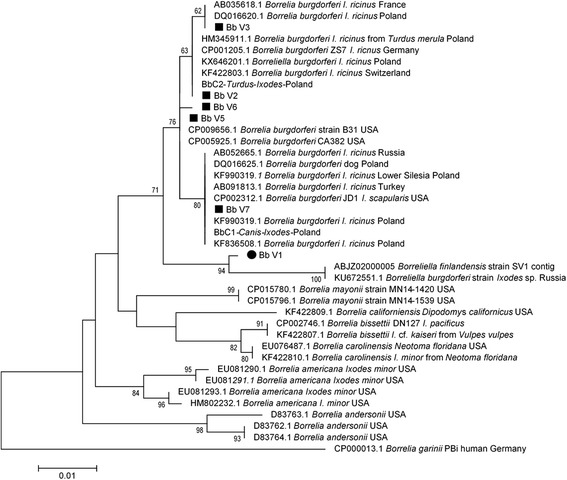
Molecular phylogenetic analysis of *flaB* variants of *B. burgdorferi* sequences obtained in the study. The phylogenetic tree was obtained with use of Maximum Likelihood method of tree construction with Tamura-Nei + G evolutionary model chosen with accordance to data by implemented model-test. The percentage of trees in which the associated taxa clustered together is shown next to the branches. The tree is drawn to scale, with branch lengths measured in the number of substitutions per site. The analysis involved 40 nucleotide sequences. There were a total of 491 positions in the final dataset. Evolutionary analyses were conducted in MEGA v. 6.06. The newly generated sequences are indicated with black symbols

Molecular phylogenetic analysis supported typing of *Borreliella* species with BLAST in all cases except one *B. burgdorferi* sequence (Additional file 5: Figure S1). Most of our sequences localised on single-species (monophyletic) branches, together with reference sequences of the same species as typed by BLAST. The *B. miyamotoi* clade rooted the *Borreliella* tree in this case. However, one sequence (547 bp) typed as *B. burgdorferi* Bb_V1 (from *I. ricinus* male, WKF, Warsaw) built a unique branch with a close relation to *B. burgdorferi* group (Additional file 5: Figure S1), forming a polyphyletic *B. burgdorferi* branch. Our variant Bb_V1 on “*B. burgdorferi* and relative species” phylogenetic tree clustered with novel European species, *B. finlandensis* (contig ABJZ02000005 and sequence KU672551), however, as a sister group (Fig. 3). The Bb_V1 sequence differed from all known *Borreliella* spp. sequences by ACG insertion. In reference to *B. finlandensis* SV1 contig 143,008–144,018 (ABJZ02000005), there were some changes in positions: 349 (G:A), 445 (T:C), 485 (T:G), 496 (G:A), 508 (A:G), 661 (C:T), an ACG-insertion in position 669–671, 688 (G:A), 712 (C:T), resulting in substitution in amino-acid sequence in position 162 (S:A) and additional glutamine (Q) amino-acid after site 222. The atypical insertion was confirmed by additional 2-repeats of sequencing in both directions with consensus sequence of 677 bp in length (MF150046). Analysis of an additional molecular marker *ospA* confirmed 100% homology of our sequence (MF150082) to ‘*B. finlandensis* Subtype 1j1 OspA partial gene’ (KM069331). On the phylogenetic tree based on the *ospA* gene fragment, our Bb_V1 sequence localised on a branch together with *Borrelia* sp. SV1 CP001524 plasmid (*B*. cf. ‘*finlandensis*’) and ‘*B. finlandensis* Subtype 1j1 OspA partial gene’ (KM069331) (Additional file 6: Figure S2).

**Fig. 4:**
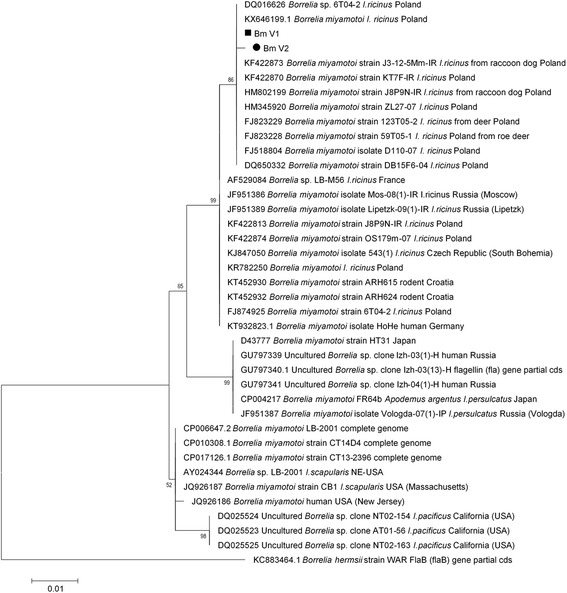
Molecular phylogenetic analysis of *B. miyamotoi flaB* sequences obtained in the study. The phylogenetic tree was obtained with use of Maximum Likelihood method of tree construction with Tamura-Nei evolutionary model chosen with accordance to data by implemented model-test. The percentage of trees in which the associated taxa clustered together is shown next to the branches. The tree is drawn to scale, with branch lengths measured in the number of substitutions per site. The analysis involved 39 nucleotide sequences. There were a total of 542 positions in the final dataset. Evolutionary analyses were conducted in MEGA v. 6.06. The newly generated sequences are indicated with black symbols

Among *B. miyamotoi flaB* sequences obtained in the study, two variants were detected from five positive ticks with direct sequencing of PCR products (5/10 positive samples): Bm_V1 and Bm_V2. Four of the *B. miyamotoi* sequences obtained were identical (Bm_V1; KT948321–3) with 100% identity with sequences from *I. ricinus* from Poland (KX646199, FJ18804). However, one sequence from WKF (Bm_V2; KT948324) differed by the one nucleotide (position 751; T:G) (99.8% similar to Bm_V1), changing the amino-acid sequence in position 249 (S:A) (ref. AY604981). On the *B. miyamotoi* phylogenetic tree based on flagellin gene fragment, our sequences clustered on Polish-origin branch of sequences, forming a separate clade (Fig. 4).

## Discussion

The main finding of our study is the discovery of similar risk of contracting tick bite and borreliosis (estimated on the basis of almost identical prevalence of spirochaetes in ticks) from two areas with opposite levels of human impact (anthropopressure). Although we compared tick abundance, prevalence and species composition of spirochaetes in *I. ricinus* ticks from distant geographically areas of Warsaw forests and park versus semi-natural forest/park sites from the vicinity of BPN, known world-wide as primeval forest habitat, we found only minor differences in tick abundance (in general, 60% higher in natural areas, but very similar in late summer-autumn period), no significant differences in the percentage of infected ticks and identical number of bacteria species (six). Despite the number of genetic variants identified within *Borreliella* species, the observed genetic differences were very minor, with the exception of one *B. burgdorferi* variant (Bb_V1), which may actually constitute a new species. Interestingly, although all spirochaetes species were found in both natural and urban sites, the analysis of common variants confirmed that only about 1/3 of *B. garinii* and *B. burgdorferi* variants were present in both areas. The majority of variants (60%) were associated with certain type of area, urban or natural.

### Abundance of ticks

Despite annual fluctuations, we observed well established tick population in urban areas, as was found in natural endemic areas. However, we observed different trends in 4-year tick abundance for these two types of areas. In urban areas the abundance oscillates on a similar level in following years. Interestingly, tick population in natural areas seemed to grow since 2013, while it was quite stable in Warsaw agglomeration. Nevertheless, mean tick abundance is over 50% higher in natural areas. Tick abundance was two times higher in natural forests compared to urban forests, which resembles findings of other studies [46, 47]. Additionally, we have registered different tick abundance between sites, which is with concordance with other studies, underlining that tick density depends on local properties of their habitat [26, 46, 48].

According to our data, total tick abundance is also generally two times higher in forests in comparison to fenced parks, despite the level of human impact in the area (natural or urban), which is similar to results of another study [22], though there is no difference in adults abundance, rather only in nymph abundance. Lower tick abundance in parks, in comparison to forests, could be explained by maintenance practice (direct anthropopressure), mowing and isolation from relatively large hosts (such as ruminants) through fencing [49–51]. Mowing restrains the forming of optimal vegetation structure (providing optimal microclimate) for ticks, while fencing limits access of large mammals, hosts for adult females, affecting tick reproduction [40, 50–53].

We also observed independent effect of *Season* on tick abundance in two types of areas: natural and urban. Generally, higher tick densities were detected in the spring-early summer season, similarly to that found in other studies [22, 54]. What is interesting is that even though tick abundance was much higher in spring-early summer season in natural areas, compared to urban areas (26.9 ± 1.9 *vs* 12.2 ± 1.2 ticks/100 m^2^) (Table 1), the mean tick abundance in late summer-autumn season was very similar in either natural or urban areas (6.1 ± 2.2 *vs* 7.9 ± 1.4 ticks/100 m^2^) (Table 1). We suspect this might be connected with host availability in spring-summer season. We assume that in natural areas the increased host availability allows more ticks to find their hosts before summer diapause, permitting the continuation of their life-cycle, resulting in similar abundance in late summer-autumn season in natural and urban areas; this concept requires further investigation.

### Prevalence of spirochaetes

We discovered almost identical prevalence (11.3%) of *Borreliella* spp. and/or *B. miyamotoi* in ticks from both types of habitats. In our study, prevalence in adults and nymphs varied from 8.4% in WBF to 18.7% in BPP, which is with agreement with results from other European and Polish studies which showed that these varied locally, from 4% to over 25% [22, 29, 44, 47, 55–60]. We have recorded significantly higher prevalence of infection with spirochaetes in *I. ricinus* ticks in second season, as opposed to recent study in the United Kingdom [22], but in agreement with the findings in the Netherlands [60]. A possible explanation of this phenomenon is a cumulative effect of transstadial transmission on prevalence of infection in *I. ricinus* during a year. There is very little possibility of transmission of these bacteria to the next generation (transovarial transmission) [61], thus together with lower tick abundance in summer-autumn, each year this cumulative effect is probably compensated for. Despite the lower abundance of ticks in the second season compared to spring-early summer, the risk of acquiring borreliosis may be still high due to this higher prevalence of infection.

Interestingly, neither the year nor the type of area has effect on the prevalence of infection with spirochaetes in our study. This risk parameter in urbanized areas was identical to endemic areas of NE Poland. It seemed that level of human impact on the area does not contribute to spirochaetes prevalence in *I. ricinus* ticks. Similar results were obtained in the USA [31].

The limitation of our study is pooling of the nymphs and use of MIR for estimation of prevalence, also for the overall prevalence in ticks. It could have resulted in lower prevalence values through ignoring possibility of more than one nymph in each pool being infected. To test this, we calculated also NIP as a measure of direct risk of infection, which assumes that more than one specimen in a pool could be infected in comparison to MIR values. However, differences between MIR and NIP were not significant. The NIP parameter was identical for both types of areas as well, meaning that the risk of acquiring the disease in case of tick bite in Warsaw urban parks and forests is equal to risk of borreliosis in endemic, low transformed forests of NE Poland.

Our findings suggest however, that in parks there might be considerably higher prevalence of *Borreliella* spp. and/or *B. miyamotoi* than in forests (18 *vs* 10%, respectively). Still, numerous European studies have shown this varies locally [29]. With almost identical prevalence of infection in ticks, and despite lower tick abundance in urban areas in spring-summer, the risk of acquiring borreliosis seems to be similar in both types of areas, as previously suggested [29]. Although there is 60% greater chance of tick encounter in natural forests, there are many more visitors in urban areas [29, 52]. Tick populations were shown to be well established in the city and the abundance of ticks is generally similar in urban and rural parks.

The overall lack of significant differences in prevalence of *Borreliella* spp. and/or *B. miyamotoi* in *I. ricinus* ticks from low and high-transformed areas is the opposite of our previous findings for tick-borne Rickettsiales. For Rickettsiales, a higher prevalence in urban habitats was explained by dilution effect [26]. However, for *Borreliella* spp. the dilution effect was recently criticized [31, 62–64]. Our findings support the lack of a dilution effect for spirochaetes prevalence and on general risk of disease appearance [65], yet further study of a potential reservoir of these bacteria in both types of areas is needed.

### Diversity of spirochaetes

Spirochaetes species richness in those two types of areas was also similar. Despite one species, *B. spielmani*, detected only once, all detected *Borreliella* spp. and *B. miyamotoi* were present in both natural and urban areas (six species). The diversity within species was minor. We found significant differences in the distribution of the species among sequenced samples. The dominant species in the study, *B. afzelii*, was also dominant in urban community (almost 70% of analyzed samples), while the second most frequent in urban areas was *B. burgdorferi.* On the other hand, in the bacteria community from natural areas, *B. garinii* was dominant (~50%), *B. afzelii* constituted only 30% and *B. burgdorferi* was less common than *B. valaisiana* (~4% *vs* ~10%, respectively). Also the diversity of *flaB* marker was greater for *B. garinii* and *B. valaisiana* in natural areas but for *B. burgdorferi* in urban areas. Both these phenomena could be explained by different host availability in both types of areas. In comparison to agglomeration-surrounded forests and city park, park and forests in low-transformed areas of NE Poland are inhabited/visited by the much higher number of host species, particularly numerous species of birds. There are 117 bird species registered in the Białowieża area, of which 90 (84%) are typical forest species [38]. On the other hand, in Warsaw, 42 bird species were recorded in parks and 70 in city forests [66], and are probably less abundant. Due to increased anthropopressure in cities, the contact of avian hosts with ticks might be limited in comparison to natural areas. Both *B. garinii* and *B. valaisiana* are associated with birds [67, 68], which also suggest the role of avian hosts in distribution of spirochaetes in natural areas, where these bacteria constitute 60% of species in positive *I. ricinus* ticks. High diversity among *B. garinii* sequences could also be explained via high rates of migration of avian hosts [69, 70]. In urban areas dominated *B. afzelii* and *B. burgdorferi*, species associated with rodent hosts, which are probably the main tick hosts and enzootic reservoir of many TBD pathogens [67]. Both larvae and nymphs of *I.ricinus* preferably feed on rodents, so there is high chance for them to get *B. afzelii* in highly urbanized areas, where rodents likely constitute the main tick hosts.

Strikingly, the ratio of *B. afzelii* in urban areas in our study is almost identical with frequency of *B. afzelii* in the Netherlands, highly urbanized and thus of high human impact region of Europe (70% of *Borreliella* spp. infections in *I. ricinus* ticks) [60]. However, also in Sweden the frequency of *B. afzelii* was estimated on similar level (61%) [71].

The presence of relapsing fever agent, *B. miyamotoi*, was limited to only two sites: BNW in Białowieża and WKF in Warsaw, and the the overall estimated prevalence and frequency were low (0.33% and 2.2%, respectively). As not all positive samples were sequenced, we cannot provide prevalence of the certain species of *Borreliella* genus or *B. miyamotoi*. Both estimated prevalence, and the ratio value of *B. miyamotoi*, are however similar to prevalence registered in other studies, varying between 0.22–3.8% [13, 22, 44, 47, 59, 71–82]. A study in Norway suggested that despite low prevalence of *B. miyamotoi*, BMD should be considered as a possible tick-borne disease [82]; we strongly agree with this conclusion. On the basis of our *B. miyamotoi* sequences, and other deposited in GenBank, we report also presence of potentially specific Polish strain of *B. miyamotoi.* Sequences of *B. miyamotoi* obtained in this study clustered with other Polish sequences on the phylogenetic tree, confirming that there is heterogeneity in flagellin sequences of European lineage of these bacteria. To date, *B. miyamotoi* is considered as conserved within 3 ‘continental’ genotypes [74, 83, 84]. We also detected a single unique variant of *B. miyamotoi* in WKF, which has no relevance currently, but further investigation might show whether it is a unique strain or aberration.

In several tick homogenates, evidence for co-existence of two species (co-infection), not only of-genus *Borreliella* but also between-genera (*Borreliella* sp. and *B. miyamotoi*), was recognized. These results raise the question of adequacy of standard PCR screening protocols and create the need for simultaneous detection and typing of different spirochaetes in ticks, e.g. via multiplex qPCR with probes. How *B. miyamotoi* interacts with *Borreliella* spp., and if such coexistence affects transmission, remains unknown and needs further scientific attention.

Finally, for the first time in Poland, we report the presence of a novel *Borreliella* sp. in *I. ricinus* tick from Warsaw Kabacki Forest. On the basis of phylogenetic analysis of *flaB* and *ospA* gene fragments we classified it as a new species related to ‘*B. finlandensis*’ (*B.* cf. ‘*finlandensis*’), as proposed by Casjens et al. [1]. Driven by unique *flaB* sequence unique features and its distinctive position in the phylogenetic tree, as well as close similarity of *ospA* sequence and formation of a separate clade with *B. finlandensis* Subtype 1j1 OspA partial gene (KM069331), we conclude that *B. finlandensis* Subtype 1j1 may in fact not belong to the ‘*B. finlandensis*’ species, but to potentially new, related species we have detected in our study. Further investigation is needed for confirmation whether our isolate is a *B. finlandensis* or a new species.

## Conclusions

There are no significant differences in *Borreliella* spp. and/or *B. miyamotoi* prevalence between low transformed, endemic areas of NE Poland and high human impact urban areas. Despite twice higher tick abundance in natural areas in spring, the mean abundance in both urban and natural areas is not dramatically different, particularly in late summer-autumn or in parks. Thus, the borreliosis risk factors appear to be similar in urban and natural areas, in cities and endemic forest areas. Analysis of *Borreliella* spp. frequency suggests that in natural areas it is more likely to develop neuroborreliosis, caused by *B. garinii*, while in urban areas there may be an increased risk of skin borreliosis, caused by *B. afzelii*. Although the prevalence of *B. miyamotoi* in ticks is relatively low it might be underestimated due to co-infections with *Borreliella* spp. in ticks and due to lower detectability. The risk of developing borreliosis or BMD seems to be similar in the city and in endemic areas in case of tick-bite, but the overall risk requires further investigation. Awareness of tick-borne spirochaetoses should be increased, in concordance with ‘One Health’ approach.

## Additional files

Additional file 1: Table S1. Ticks abundance in two subtypes of area: forests and parks (mean ± SE). (DOCX 19 kb)

Additional file 2: Table S2. *Ixodes ricinus* nymph abundance in natural and urban areas (mean ± SE). (DOCX 17 kb)

Additional file 3: Table S3. Abundance of *I. ricinus* females and males in natural and urban areas (mean ± SE). (DOCX 21 kb)

Additional file 4: Table S4. Statistical table of ANOVA (GLM) analysis of tick abundance and ML HILOGLINEAR analyses of spirochaetes prevalence and distribution. (DOCX 17 kb)

Additional file 5: Figure S1. Molecular phylogenetic analysis of *flaB* variants of sequences obtained in the study. The phylogenetic tree was obtained with use of Maximum Likelihood method of tree construction with Tamura-Nei + G evolutionary model chosen with accordance to data by implemented model-test. The percentage of trees in which the associated taxa clustered together is shown next to the branches. The tree is drawn to scale, with branch lengths measured in the number of substitutions per site. The analysis involved 94 nucleotide sequences. There were a total of 547 positions in the final dataset. Evolutionary analyses were conducted in MEGA v. 6.06. The newly generated sequences and their origin (type of area) are indicated with black symbols: triangle, natural areas; upside down tringle, urban areas; diamond, both natural and urban areas. (DOCX 1021 kb)

Additional file 6: Figure S2. Molecular phylogenetic analysis of Bb_V1 *ospA* fragment. The phylogenetic tree was obtained with use of Maximum Likelihood method of tree construction with Tamura-Nei + G evolutionary model chosen with accordance to data by implemented model-test. The percentage of trees in which the associated taxa clustered together is shown next to the branches. The tree is drawn to scale, with branch lengths measured in the number of substitutions per site. The analysis involved 19 nucleotide sequences. There were a total of 652 positions in the final dataset. Evolutionary analyses were conducted in MEGA v. 6.06. The newly generated sequences are indicated with black symbols. Underneath the main tree is visualized zoomed *‘B. finlandensis’* branch. (DOCX 475 kb)

## Abbreviations

BMD: ‘*Borrelia miyamotoi* disease’
BNP: Białowieża National Park;
BNW: Białowieża, North-West
BPP: Białowieża, Palace Park
BSW: Białowieża, South-West
CI: Confidence intervals
LD: Lyme disease
RF: Relapsing fever
WBF: Warsaw, Bielański Forest
WKF: Warsaw, Kabacki Forest
WLP: Warsaw, Łazienki Park
