# Supplementary material for: Ticks and the city - are there any differences between city parks and natural forests in terms of tick abundance and prevalence of spirochaetes?
Source: Parasit Vectors. 2017 Nov 21;10:573. doi: 10.1186/s13071-017-2391-2 (PMC5697153; doi:10.1186/s13071-017-2391-2)
Supplement: Supplementary file 1 — Ticks abundance in two subtypes of area: forests and parks (mean ± SE). (DOCX 19 kb) [file 13071_2017_2391_MOESM1_ESM.docx]

| **Additional file 1: Table S1.** Ticks abundance in two subtypes of area: forests and parks (mean ± SE) | | | | | | | | | | | | | | | | | | | | | |
| --- | --- | --- | --- | --- | --- | --- | --- | --- | --- | --- | --- | --- | --- | --- | --- | --- | --- | --- | --- | --- | --- |
|  | |  | **Total tick abundance** | | | |  | **Mean females abundance (Mean + SE)** | | | |  | **Mean males abundance (Mean + SE)** | | | |  | **Mean nymph abundance (Mean + SE)** | | | |
|  |  |  | **Subtype** | |  | **Natural + Urban** |  | **Subtype of area** | |  | **Natural + Urban** |  | **Subtype of area** | |  | **Natural + Urban** |  | **Subtype of area** | |  | **Natural + Urban** |
| **Year** | **Season** |  | **Forest (1)** | **Park (2)** |  | **Mean (1+2)** |  | **Forest (1)** | **Park (2)** |  | **Mean (1+2)** |  | **Forest (1)** | **Park (2)** |  | **Mean (1+2)** |  | **Forest (1)** | **Park (2)** |  | **Mean (1+2)** |
| **2012** | **1** |  | 20.8 ± 2.3 | 12.6 ± 6 |  | 16.7 ± 3.2 |  | 4 ± 0.7 | 5.2 ± 1.7 |  | 4.6 ± 0.9 |  | 4.7 ± 0.7 | 5 ± 1.8 |  | 4.8 ± 1 |  | 12.1 ± 1.4 | 2.4 ± 3.5 |  | 7.2 ± 1.9 |
|  | **2** |  | 5.1 ± 2.6 | 0.3 ± 7.8 |  | 2.7 ± 4.1 |  | 0.6 ± 0.7 | 0.3 ± 2.2 |  | 0.5 ± 1.2 |  | 1 ± 0.8 | 0 ± 2.4 |  | 0.5 ± 1.2 |  | 3.5 ± 1.5 | 0 ± 4.6 |  | 1.7 ± 2.4 |
|  | **Total** |  | **12.9 ± 1.7** | **6.5 ± 4.9** |  | **9.7 ± 2.6** |  | **2.3 ± 0.5** | **2.8 ± 1.4** |  | **2.5 ± 0.7** |  | **2.8 ± 0.5** | **2.5 ± 1.5** |  | **2.7 ± 0.8** |  | **7.8 ± 1** | **1.2 ± 2.9** |  | **4.5 ± 1.5** |
| **2013** | **1** |  | 15.5 ± 2.4 | 9.4 ± 3.4 |  | 12.5 ± 2.1 |  | 2.7 ± 0.7 | 2.6 ± 1 |  | 2.6 ± 0.6 |  | 2.8 ± 0.7 | 2.2 ± 1 |  | 2.5 ± 0.6 |  | 10 ± 1.4 | 4.7 ± 2 |  | 7.3 ± 1.2 |
|  | **2** |  | 10.2 ± 2.5 | 4.6 ± 3.6 |  | 7.4 ± 2.2 |  | 1.4 ± 0.7 | 2.1 ± 1 |  | 1.8 ± 0.6 |  | 1 ± 0.8 | 1.2 ± 1.1 |  | 1.1 ± 0.7 |  | 7.8 ± 1.5 | 1.2 ± 2.1 |  | 4.5 ± 1.3 |
|  | **Total** |  | **12.9 ± 1.7** | **7 ± 2.5** |  | **10 ± 1.5** |  | **2 ± 0.5** | **2.4 ± 0.7** |  | **2.2 ± 0.4** |  | **1.9 ± 0.5** | **1.7 ± 0.7** |  | **1.8 ± 0.5** |  | **8.9 ± 1** | **3 ± 1.4** |  | **5.9 ± 0.9** |
| **2014** | **1** |  | 14.6 ± 2.5 | 10.1 ± 3.6 |  | 12.4 ± 2.2 |  | 4.5 ± 0.7 | 4.3 ± 1 |  | 4.4 ± 0.6 |  | 5.6 ± 0.7 | 4.4 ± 1.1 |  | 5 ± 0.7 |  | 4.6 ± 1.4 | 1.5 ± 2.1 |  | 3 ± 1.3 |
|  | **2** |  | 7.2 ± 2.5 | 2.4 ± 4.2 |  | 4.8 ± 2.5 |  | 2.8 ± 0.7 | 1.3 ± 1.2 |  | 2.1 ± 0.7 |  | 2.5 ± 0.8 | 1 ± 1.3 |  | 1.7 ± 0.8 |  | 1.9 ± 1.5 | 0.1 ± 2.5 |  | 1 ± 1.5 |
|  | **Total** |  | **10.9 ± 1.8** | **6.3 ± 2.8** |  | **8.6 ± 1.6** |  | **3.6 ± 0.5** | **2.8 ± 0.8** |  | **3.2 ± 0.5** |  | **4 ± 0.5** | **2.7 ± 0.8** |  | **3.4 ± 0.5** |  | **3.2 ± 1** | **0.8 ± 1.6** |  | **2 ± 1** |
| **2015** | **1** |  | 22.2 ± 2.5 | 11.9 ± 4.2 |  | 17 ± 2.5 |  | 5.4 ± 0.7 | 5 ± 1.2 |  | 5.2 ± 0.7 |  | 6.4 ± 0.8 | 4.1 ± 1.3 |  | 5.3 ± 0.7 |  | 10.4 ± 1.5 | 2.8 ± 2.5 |  | 6.6 ± 1.4 |
|  | **2** |  | 14.3 ± 3.9 | 2.8 ± 6 |  | 8.5 ± 3.6 |  | 3.3 ± 1.1 | 1.6 ± 1.7 |  | 2.5 ± 1 |  | 3 ± 1.2 | 0.8 ± 1.8 |  | 1.9 ± 1.1 |  | 7.9 ± 2.3 | 0.4 ± 3.5 |  | 4.2 ± 2.1 |
|  | **Total** |  | **18.2 ± 2.3** | **7.3 ± 3.7** |  | **12.8 ± 2.2** |  | **4.4 ± 0.7** | **3.3 ± 1.1** |  | **3.8 ± 0.6** |  | **4.7 ± 0.7** | **2.4 ± 1.1** |  | **3.6 ± 0.7** |  | **9.2 ± 1.4** | **1.6 ± 2.2** |  | **5.4 ± 1.3** |
| **4-year mean** | **1** |  | 18.3 ± 1.2 | 11 ± 2.2 |  | 14.6 ± 1.3 |  | 4.2 ± 0.3 | 4.3 ± 0.6 |  | 4.2 ± 0.4 |  | 4.9 ± 0.4 | 3.9 ± 0.7 |  | 4.4 ± 0.4 |  | 9.3 ± 0.7 | 2.8 ± 1.3 |  | 6.1 ± 0.7 |
|  | **2** |  | 9.2 ± 1.5 | 2.5 ± 2.8 |  | 5.9 ± 1.6 |  | 2 ± 0.4 | 1.3 ± 0.8 |  | 1.7 ± 0.5 |  | 1.9 ± 0.4 | 0.8 ± 0.9 |  | 1.3 ± 0.5 |  | 5.3 ± 0.9 | 0.4 ± 1.7 |  | 2.8 ± 0.9 |
|  | **Total** |  | **13.7 ± 1** | **6.8 ± 1.8** |  | **10.3 ± 1** |  | **3.1 ± 0.3** | **2.8 ± 0.5** |  | **3 ± 0.3** |  | **3.4 ± 0.3** | **2.3 ± 0.5** |  | **2.9 ± 0.3** |  | **7.3 ± 0.6** | **1.6 ± 1.1** |  | **4.5 ± 0.6** |

Legend: Season (1 = first. spring-early summer; 2 = late summer-autumn); Subtype (1 = forest; 2 = park)
