# Supplementary material for: Ticks and the city - are there any differences between city parks and natural forests in terms of tick abundance and prevalence of spirochaetes?
Source: Parasit Vectors. 2017 Nov 21;10:573. doi: 10.1186/s13071-017-2391-2 (PMC5697153; doi:10.1186/s13071-017-2391-2)
Supplement: Supplementary file 2 — Ixodes ricinus nymph abundance in natural and urban areas (mean ± SE). (DOCX 17 kb) [file 13071_2017_2391_MOESM2_ESM.docx]

| **Additional file 2: Table S2.** *Ixodes ricinus* nymph abundance in natural and urban areas (mean ± SE) | | | | | | | | | | | | | |
| --- | --- | --- | --- | --- | --- | --- | --- | --- | --- | --- | --- | --- | --- |
|  | |  | **Mean tick abundance (Mean + SE)** | | | | | | | | | | |
|  |  |  | **Type of area/ Site** | | | | | | | | | | |
|  |  |  | **Subtype/Natural areas** | | | |  | **Subtype/Urban areas** | | | |  | **Natural + Urban** |
|  |  |  | **1** | **1** | **2** | **1+2** |  | **1** | **1** | **2** | **1+2** |  | **1+2** |
| **Year** | **Season** |  | **Białowieża North-West** | **Białowieża South-West** | **Białowieża Palace Park** | **Mean Natural** |  | **Bielański Forest** | **Kabacki Forest** | **Royal Łazienki Park** | **Mean Urban** |  | **Mean** |
| **2012** | **1** |  | 27.9 ± 2.1 | 8 ± 3.1 | 2.4 ± 2.8 | 16.4 ± 1.8 |  | 5.3 ± 2 | 6.3 ± 2 | ND | 5.8 ± 1.7 |  | 11.1 ± 1.3 |
|  | **2** |  | 3.7 ± 3.6 | 1.7 ± 3.6 | 0 ± 3.6 | 1.8 ± 2.6 |  | 5.9 ± 1.8 | 0.8 ± 2.1 | ND | 3.7 ± 1.7 |  | 2.7 ± 1.5 |
|  | **Total** |  | **15.8 ± 2.1** | **4.8 ± 2.4** | **1.2 ± 2.3** | **9.1 ± 1.6** |  | **5.6 ± 1.3** | **3.5 ± 1.4** | **ND** | **4.8 ± 1.2** |  | **6.9 ± 1** |
| **2013** | **1** |  | 3.8 ± 2.8 | 1.2 ± 2.8 | 0.6 ± 2.8 | 1.9 ± 2 |  | 19.3 ± 1.9 | 7.3 ± 2 | 6.5 ± 1.9 | 11.2 ± 1.4 |  | 6.5 ± 1.2 |
|  | **2** |  | 7.5 ± 4.4 | 1.5 ± 4.4 | 2.5 ± 4.4 | 3.8 ± 3.2 |  | 10.1 ± 1.7 | 6.4 ± 1.8 | 1 ± 1.8 | 5.9 ± 1.3 |  | 4.9 ± 1.7 |
|  | **Total** |  | **5.6 ± 2.6** | **1.4 ± 2.6** | **1.6 ± 2.6** | **2.9 ± 1.9** |  | **14.7 ± 1.3** | **6.9 ± 1.3** | **3.8 ± 1.3** | **8.6 ± 0.9** |  | **5.7 ± 1.1** |
| **2014** | **1** |  | 13.3 ± 3.1 | 7.8 ± 3.1 | 0.5 ± 3.1 | 7.2 ± 2.2 |  | 1.2 ± 1.9 | 3.6 ± 1.9 | 1.9 ± 2 | 2.3 ± 1.4 |  | 4.7 ± 1.3 |
|  | **2** |  | 2.3 ± 3.1 | 1.2 ± 3.1 | 0 ± 4.4 | 1.4 ± 2.5 |  | 1.5 ± 2 | 2.3 ± 2 | 0.1 ± 2.2 | 1.4 ± 1.5 |  | 1.4 ± 1.4 |
|  | **Total** |  | **7.8 ± 2.2** | **4.5 ± 2.2** | **0.2 ± 2.7** | **4.3 ± 1.7** |  | **1.3 ± 1.4** | **3 ± 1.4** | **1 ± 1.5** | **1.8 ± 1** |  | **3.1 ± 1** |
| **2015** | **1** |  | 36 ± 4.4 | 6 ± 4.4 | 0.5 ± 4.4 | 14.2 ± 3.2 |  | 12.7 ± 1.7 | 4.4 ± 1.8 | 3.4 ± 2.2 | 7.4 ± 1.4 |  | 10.8 ± 1.7 |
|  | **2** |  | 13.5 ± 4.4 | 1.5 ± 4.4 | 0.5 ± 4.4 | 5.2 ± 3.2 |  | 11.3 ± 3.1 | 5 ± 3.1 | 0.3 ± 3.6 | 6 ± 2.3 |  | 5.6 ± 2 |
|  | **Total** |  | **24.8 ± 3.1** | **3.7 ± 3.1** | **0.5 ± 3.1** | **9.7 ± 2.2** |  | **12 ± 1.8** | **4.7 ± 1.8** | **1.9 ± 2.1** | **6.7 ± 1.4** |  | **8.2 ± 1.3** |
| **4-year mean** | **1** |  | 20.2 ± 1.6 | 5.7 ± 1.7 | 1 ± 1.7 | 9.9 ± 1.2 |  | 9.6 ± 0.9 | 5.4 ± 1 | 3.9 ± 1.2 | 6.7 ± 0.7 |  | 8.3 ± 0.7 |
|  | **2** |  | 6.7 ± 2 | 1.5 ± 2 | 0.7 ± 2.1 | 3 ± 1.4 |  | 7.2 ± 1.1 | 3.6 ± 1.2 | 0.5 ± 1.5 | 4.3 ± 0.9 |  | 3.7 ± 0.8 |
|  | **Total** |  | **13.5 ± 1.3** | **3.6 ± 1.3** | **0.9 ± 1.3** | **6.5 ± 0.9** |  | **8.4 ± 0.7** | **4.5 ± 0.7** | **2.2 ± 1** | **5.5 ± 0.6** |  | **6 ± 0.5** |

Legend: Season (1 = first, spring-early summer; 2 = late summer-autumn); Subtype (1 = forest; 2 = park)
