# Supplementary material for: Ticks and the city - are there any differences between city parks and natural forests in terms of tick abundance and prevalence of spirochaetes?
Source: Parasit Vectors. 2017 Nov 21;10:573. doi: 10.1186/s13071-017-2391-2 (PMC5697153; doi:10.1186/s13071-017-2391-2)
Supplement: Supplementary file 3 — Abundance of I. ricinus females and males in natural and urban areas (mean ± SE). (DOCX 21 kb) [file 13071_2017_2391_MOESM3_ESM.docx]

| **Additional file 3: Table S3.** Abundance of *I. ricinus* females and males in natural and urban areas (mean ± SE). | | | | | | | | | | | | | | | | | | | | | |  | |  |
| --- | --- | --- | --- | --- | --- | --- | --- | --- | --- | --- | --- | --- | --- | --- | --- | --- | --- | --- | --- | --- | --- | --- | --- | --- |
|  | |  | **Mean tick abundance (Mean + SE)** | | | | | | | | | | | | | | | | | | |  | |  |
|  |  |  | **Type of area/ Site** | | | | | | | | | | | | | | | | | | | | |  |
|  |  |  | **Subtype/Natural areas/Sex** | | | | | | | |  | **Subtype/Urban areas/Sex** | | | | | | | |  | **Natural + Urban** | | | |
|  |  |  | **1** | | **1** | | **2** | | **1+2** | |  | **1** | | **1** | | **2** | | **1+2** | |  | **1+2** | | | |
| **Year** | **Season** |  | **Białowieża North-West** | | **Białowieża South-West** | | **Białowieża Palace Park** | | **Mean Natural** | |  | **Bielański Forest** | | **Kabacki Forest** | | **Royal Łazienki Park** | | **Mean Urban** | |  | **Mean** | | | |
|  |  |  | **Female** | **Male** | **Female** | **Male** | **Female** | **Male** | **Female** | **Male** |  | **Female** | **Male** | **Female** | **Male** | **Female** | **Male** | **Female** | **Male** |  | **Female** | | **Male** | |
| **2012** | **1** |  | 9 ± 1 | 10.8 ± 1 | 10 ± 1.5 | 8.8 ± 1.5 | 5.2 ± 1.3 | 5 ± 1.3 | 8.2 ± 0.8 | 8.7 ± 0.8 |  | 0.6 ± 0.9 | 1.3 ± 0.9 | 0.6 ± 0.9 | 0.9 ± 0.9 | ND | ND | 0.6 ± 0.7 | 1.1 ± 0.7 |  | 4.4 ± 0.5 | | 4.9 ± 0.5 | |
|  | **2** |  | 1 ± 1.7 | 0.7 ± 1.7 | 1 ± 1.7 | 2.3 ± 1.7 | 0.3 ± 1.7 | 0 ± 1.7 | 0.8 ± 1.1 | 1 ± 1.1 |  | 0.7 ± 0.8 | 1.1 ± 0.9 | 0.2 ± 1 | 0.6 ± 1 | ND | ND | 0.5 ± 0.7 | 0.9 ± 0.7 |  | 0.6 ± 0.6 | | 0.9 ± 0.7 | |
|  | **Total** |  | **5 ± 1** | **5.7 ± 1** | **5.5 ± 1.1** | **5.5 ± 1.1** | **2.8 ± 1.1** | **2.5 ± 1.1** | **4.5 ± 0.7** | **4.9 ± 0.7** |  | **0.6 ± 0.6** | **1.2 ± 0.6** | **0.4 ± 0.7** | **0.7 ± 0.7** | **ND** | **ND** | **0.5 ± 0.5** | **1 ± 0.5** |  | **2.5 ± 0.4** | | **2.9 ± 0.4** | |
| **2013** | **1** |  | 2 ± 1.3 | 2.2 ± 1.3 | 2.8 ± 1.3 | 3.2 ± 1.3 | 1.4 ± 1.3 | 0.4 ± 1.3 | 2.1 ± 0.8 | 1.9 ± 0.9 |  | 3 ± 0.9 | 3.1 ± 0.9 | 2.7 ± 0.9 | 2.5 ± 0.9 | 3.1 ± 0.9 | 3 ± 0.9 | 2.9 ± 0.6 | 2.9 ± 0.6 |  | 2.5 ± 0.5 | | 2.4 ± 0.5 | |
|  | **2** |  | 1 ± 2.1 | 1 ± 2.1 | 2 ± 2.1 | 2 ± 2.1 | 0 ± 2.1 | 0.5 ± 2.1 | 1 ± 1.3 | 1.2 ± 1.4 |  | 1.2 ± 0.8 | 0.9 ± 0.8 | 1.6 ± 0.8 | 1 ± 0.9 | 2.5 ± 0.8 | 1.3 ± 0.9 | 1.7 ± 0.5 | 1.1 ± 0.5 |  | 1.4 ± 0.7 | | 1.1 ± 0.7 | |
|  | **Total** |  | **1.5 ± 1.2** | **1.6 ± 1.3** | **2.4 ± 1.2** | **2.6 ± 1.3** | **0.7 ± 1.2** | **0.5 ± 1.3** | **1.5 ± 0.8** | **1.6 ± 0.8** |  | **2.1 ± 0.6** | **2 ± 0.6** | **2.1 ± 0.6** | **1.8 ± 0.6** | **2.8 ± 0.6** | **2.2 ± 0.6** | **2.3 ± 0.4** | **2 ± 0.4** |  | **1.9 ± 0.4** | | **1.8 ± 0.5** | |
| **2014** | **1** |  | 9.5 ± 1.5 | 10.5 ± 1.5 | 8.3 ± 1.5 | 12 ± 1.5 | 4 ± 1.5 | 4.8 ± 1.5 | 7.3 ± 0.9 | 9.1 ± 1 |  | 4.1 ± 0.9 | 5.4 ± 0.9 | 1.6 ± 0.9 | 1.7 ± 0.9 | 4.4 ± 0.9 | 4.2 ± 0.9 | 3.3 ± 0.6 | 3.8 ± 0.6 |  | 5.3 ± 0.5 | | 6.4 ± 0.6 | |
|  | **2** |  | 2.5 ± 1.5 | 2 ± 1.5 | 0.5 ± 1.5 | 0.2 ± 1.5 | 1.5 ± 2.1 | 0.5 ± 2.1 | 1.5 ± 1 | 1 ± 1.1 |  | 5 ± 0.9 | 4.1 ± 0.9 | 1.7 ± 0.9 | 1.9 ± 0.9 | 1.3 ± 1 | 1.1 ± 1.1 | 2.8 ± 0.6 | 2.5 ± 0.6 |  | 2.1 ± 0.6 | | 1.7 ± 0.6 | |
|  | **Total** |  | **6 ± 1** | **6.3 ± 1.1** | **4.4 ± 1** | **6.1 ± 1.1** | **2.8 ± 1.3** | **2.6 ± 1.3** | **4.4 ± 0.7** | **5 ± 0.7** |  | **4.5 ± 0.6** | **4.7 ± 0.7** | **1.7 ± 0.6** | **1.8 ± 0.7** | **2.8 ± 0.7** | **2.7 ± 0.7** | **3 ± 0.4** | **3.1 ± 0.4** |  | **3.7 ± 0.4** | | **4.1 ± 0.4** | |
| **2015** | **1** |  | 24 ± 2.1 | 26 ± 2.1 | 15 ± 2.1 | 19 ± 2.1 | 3 ± 2.1 | 5 ± 2.1 | 14 ± 1.3 | 16.7 ± 1.4 |  | 3.7 ± 0.8 | 3.9 ± 0.8 | 2.7 ± 0.8 | 3.8 ± 0.9 | 5.5 ± 1 | 3.9 ± 1.1 | 3.8 ± 0.6 | 3.8 ± 0.6 |  | 8.9 ± 0.7 | | 10.3 ± 0.7 | |
|  | **2** |  | 4.5 ± 2.1 | 2 ± 2.1 | 4 ± 2.1 | 4.5 ± 2.1 | 1.5 ± 2.1 | 0.5 ± 2.1 | 3.3 ± 1.3 | 2.3 ± 1.4 |  | 2.5 ± 1.5 | 3.8 ± 1.5 | 3.2 ± 1.5 | 2 ± 1.5 | 1.7 ± 1.7 | 1 ± 1.7 | 2.5 ± 1 | 2.4 ± 1 |  | 2.9 ± 0.8 | | 2.3 ± 0.8 | |
|  | **Total** |  | **14.3 ± 1.5** | **14 ± 1.5** | **9.5 ± 1.5** | **11.8 ± 1.5** | **2.3 ± 1.5** | **2.8 ± 1.5** | **8.7 ± 0.9** | **9.5 ± 1** |  | **3.1 ± 0.8** | **3.8 ± 0.9** | **3 ± 0.8** | **2.9 ± 0.9** | **3.6 ± 1** | **2.4 ± 1** | **3.2 ± 0.6** | **3.1 ± 0.6** |  | **5.9 ± 0.5** | | **6.3 ± 0.6** | |
| **4-year mean** | **1** |  | 11.1 ± 0.8 | 12.4 ± 0.8 | 9 ± 0.8 | 10.7 ± 0.8 | 3.4 ± 0.8 | 3.8 ± 0.8 | 7.9 ± 0.5 | 9.1 ± 0.5 |  | 2.8 ± 0.4 | 3.4 ± 0.4 | 1.9 ± 0.4 | 2.2 ± 0.5 | 4.3 ± 0.5 | 3.7 ± 0.6 | 2.7 ± 0.3 | 2.9 ± 0.3 |  | 5.3 ± 0.3 | | 6 ± 0.3 | |
|  | **2** |  | 2.2 ± 0.9 | 1.4 ± 0.9 | 1.9 ± 0.9 | 2.3 ± 0.9 | 0.8 ± 1 | 0.4 ± 1 | 1.7 ± 0.6 | 1.4 ± 0.6 |  | 2.3 ± 0.5 | 2.5 ± 0.5 | 1.7 ± 0.5 | 1.4 ± 0.6 | 1.8 ± 0.7 | 1.2 ± 0.7 | 1.9 ± 0.4 | 1.7 ± 0.4 |  | 1.8 ± 0.3 | | 1.5 ± 0.4 | |
|  | **Total** |  | **6.7 ± 0.6** | **6.9 ± 0.6** | **5.4 ± 0.6** | **6.5 ± 0.6** | **2.1 ± 0.6** | **2.1 ± 0.6** | **4.8 ± 0.4** | **5.2 ± 0.4** |  | **2.6 ± 0.3** | **2.9 ± 0.3** | **1.8 ± 0.4** | **1.8 ± 0.4** | **3.1 ± 0.5** | **2.4 ± 0.5** | **2.3 ± 0.2** | **2.3 ± 0.2** |  | **3.5 ± 0.2** | | **3.8 ± 0.2** | |

Legend: Season (1 = first, spring-early summer; 2 = late summer-autumn); Subtype (1 = forest; 2 = park)
