# Supplementary material for: Ticks and the city - are there any differences between city parks and natural forests in terms of tick abundance and prevalence of spirochaetes?
Source: Parasit Vectors. 2017 Nov 21;10:573. doi: 10.1186/s13071-017-2391-2 (PMC5697153; doi:10.1186/s13071-017-2391-2)
Supplement: Supplementary file 4 — Statistical table of ANOVA (GLM) analysis of tick abundance and ML HILOGLINEAR analyses of spirochaetes prevalence and distribution. (DOCX 17 kb) [file 13071_2017_2391_MOESM4_ESM.docx]

**Additional file 4: Table S4.** Statistical table of ANOVA (GLM) analysis of tick abundance and ML HILOGLINEAR analyses of spirochaetes prevalence and distribution

| **Tick abundance** | | |
| --- | --- | --- |
| Population | **General Linear Model (GLM)** | **Effects and effect interactions statistics** |
| **Total (females + males + nymphs)** | **Year × Season × Type of area** | Main effect of Year: F_3, 295_ = 6.9,  *P* <0.001 |
|  |  | Main effect of Season: F_1, 295_  = 57.3,  *P* <0.001 |
|  |  | Main effect of Type of area: F_1, 295_ = 15.2,  *P* < 0.001 |
|  |  | Effect interaction of Year × Season: F_1, 295_  = 3.7,  *P* = 0.012 |
|  |  | Effect interaction of Year × Type of area: F_3, 295_ = 8.4,  *P* < 0.001 |
|  |  | Effect interaction of Season × Type of area: F_1, 295_ = 24.7,  *P* < 0.001 |
|  |  | Effect interaction of Year × Season × Type of area: F_1, 295_ = 6.9,  *P* < 0.001 |
|  | **Year × Season × Site** | Main effect of Site: F_5, 295_ = 18.9,  *P* < 0.001 |
|  |  | Effect interaction of Year × Site F_14, 295_ = 4.5,  *P* < 0.001 |
|  |  | Effect interaction of Season × Site: F_5, 295_ = 11.3,  *P* < 0.001 |
|  |  | Effect interaction of Year × Season × Site: F_14, 295_ = 3.3,  *P* < 0.001. |
|  | **Year × Season × Subtype of area** | Main effect of Season: F_1, 295_ = 18.8,  *P* < 0.001 |
|  |  | Main effect of SubType of area: F_1, 295_ = 11.7,  *P* = 0.001 |
|  |  | other effects or effect interactions: NS |
| **Nymphs** | **Year × Season × Type of area** | Main effect of Year: F_3, 295_ = 4.2,  *P* = 0.07 |
|  |  | Main effect of Season: F_1, 295_ = 17.9,  *P* < 0.001 |
|  |  | Main effect of Type of area: NS Main effect of Year × Season: NS |
|  |  | Effect interaction of Year × Type of area: F_3, 295_ = 4.7,  *P* = 0.003 |
|  |  | Effect interaction of Season × Type of area: F_1, 295_ = 4.2,  *P* = 0.042 |
|  |  | Effect interaction of Year × Season × Type of area: F_3, 295_ = 4.0,  *P* = 0.008 |
|  | **Year × Season × Site** | Main effect of: Site: F_5, 295_ = 16.1,  *P* < 0.001 |
|  |  | Effect interaction of Year × Site: F_14, 295_ = 4.5,  *P* < 0.001 |
|  |  | Effect interaction of Season × Site: F_5, 295_ = 3.8,  *P* = 0.002 |
|  |  | Effect interaction of Year × Season × Site: F_14, 295_ = 2.2,  *P* = 0.007. |
|  | **Year × Season × Subtype of area** | Main effect of Year: F_3, 295_ = 3.2,  *P* = 0.02 |
|  |  | Main effect of Season: F_1, 295_ = 7.2,  *P* = 0.008 |
|  |  | Main effect of Subtype of area: F_1, 295_ = 22.2,  *P* < 0.001 |
|  |  | other effects or effect interactions: NS. |
| **Females** | **Year × Season × Type of area** | Main effect of Year: F_3, 295_ = 12.5,  *P* < 0.001 |
|  |  | Main effect of Season: F_1, 295_ = 59.6,  *P* < 0.001 |
|  |  | Main effect of Type of area: F_1, 295_ = 30.2,  *P* < 0.001 |
|  |  | Effect interaction of Year × Season: F_3, 295_ = 4.1,  *P* = 0.007 |
|  |  | Effect interaction of Year × Type of area: F_3, 295_ = 8.9,  *P* < 0.001 |
|  |  | Effect interaction of Season × Type of area: F_1, 295_ = 35.9,  *P* < 0.001 |
|  |  | Effect interaction of Year × Season × Type of area: F_3, 295_ = 4.9,  *P* = 0.002 |
|  | **Year × Season × Site** | Main effect of Site: F_5, 295_ = 14.0,  *P* < 0.001 |
|  |  | Effect interaction of Year × Site F_14, 295_ = 4.2,  *P* < 0.001 |
|  |  | Effect interaction of Season × Site: F_5, 295_ = 12.5,  *P* < 0.001 |
|  |  | Effect interaction of Year × Season × Site: F_14, 295_ = 2.6,  *P* = 0.002 |
|  | **Year × Season × SubType of area** | Main effect of Season: F_1, 295_ = 19.0,  *P* = < 0.001 |
|  |  | other effects or effect interactions: NS. |
| **Males** | **Year × Season × Type of area** | Main effect of Year: F_3, 295_ = 14.5,  *P* < 0.001 |
|  |  | Main effect of Season F_1, 295_  = 91.0,  *P* < 0.001 |
|  |  | Main effect of Type of area: F_1, 295_ = 39.6,  *P* < 0.001 |
|  |  | Effect interaction of Year × Season: F_3, 295_ = 7.3,  *P* < 0.001 |
|  |  | Effect interaction of Year × Type of area: F_3, 295_ = 8.5,  *P* < 0.001 |
|  |  | Effect interaction of Season × Type of area: F_1, 295_ = 48.6,  *P* <0.001 |
|  |  | Effect interaction of Year × Season × Type of area: F_3, 295_ = 8.5,  *P* < 0.001 |
|  | **Year × Season × Site** | Main effect of Site: F_5, 295_  = 17.6,  *P* < 0.001 |
|  |  | Effect interaction of Year × Site: F_14, 295_ = 3.8,  *P* < 0.001 |
|  |  | Effect interaction of Season × Site: F_5, 295_ = 16.0,  *P* < 0.001 |
|  |  | Effect interaction of Year × Season × Site: F_14, 295_ = 3.5,  *P* < 0.001 |
|  | **Year × Season × Subtype of area** | Main effect of Season: F_1, 295_  = 24.8,  *P* < 0.001 |
|  |  | other effects or effect interactions: NS |
| **Prevalence of *Borreliella* spp. and/or *Borrelia miyamotoi* spirochaetes in ticks** | | |
|  | **Hierarchic logline analysis** | Backward elimination statistics |
|  | **Season × presence/absence of *Borreliella* spp. and/or *B. miyamotoi*** | χ^2^ = 4.3, df = 1,  *P* = 0.039 |
|  | **Subtype of area × presence/absence of *Borreliella* spp. and/or *B. miyamotoi*** | χ2= 7.6; df= 1; *P* = 0.006 |
| **Distribution (frequency) of *Borreliella* spp. and *B. miyamotoi*** | | |
|  | **Hierarchic logline analysis** | Backward elimination statistics |
|  | **Type of area × species** | χ^2^= 67.6; df = 6; *P* < 0.001 |
|  | **Type of area × common species** | χ^2^= 0.3; df = 1; *P* = 0.595 |
|  | **Subtype of area × species** | χ^2^= 16.6; df = 6; *P* = 0.011 |
