# Supplementary material for: Ticks and the city - are there any differences between city parks and natural forests in terms of tick abundance and prevalence of spirochaetes?
Source: Parasit Vectors. 2017 Nov 21;10:573. doi: 10.1186/s13071-017-2391-2 (PMC5697153; doi:10.1186/s13071-017-2391-2)
Supplement: Supplementary file 5 [file 13071_2017_2391_MOESM5_ESM.docx]

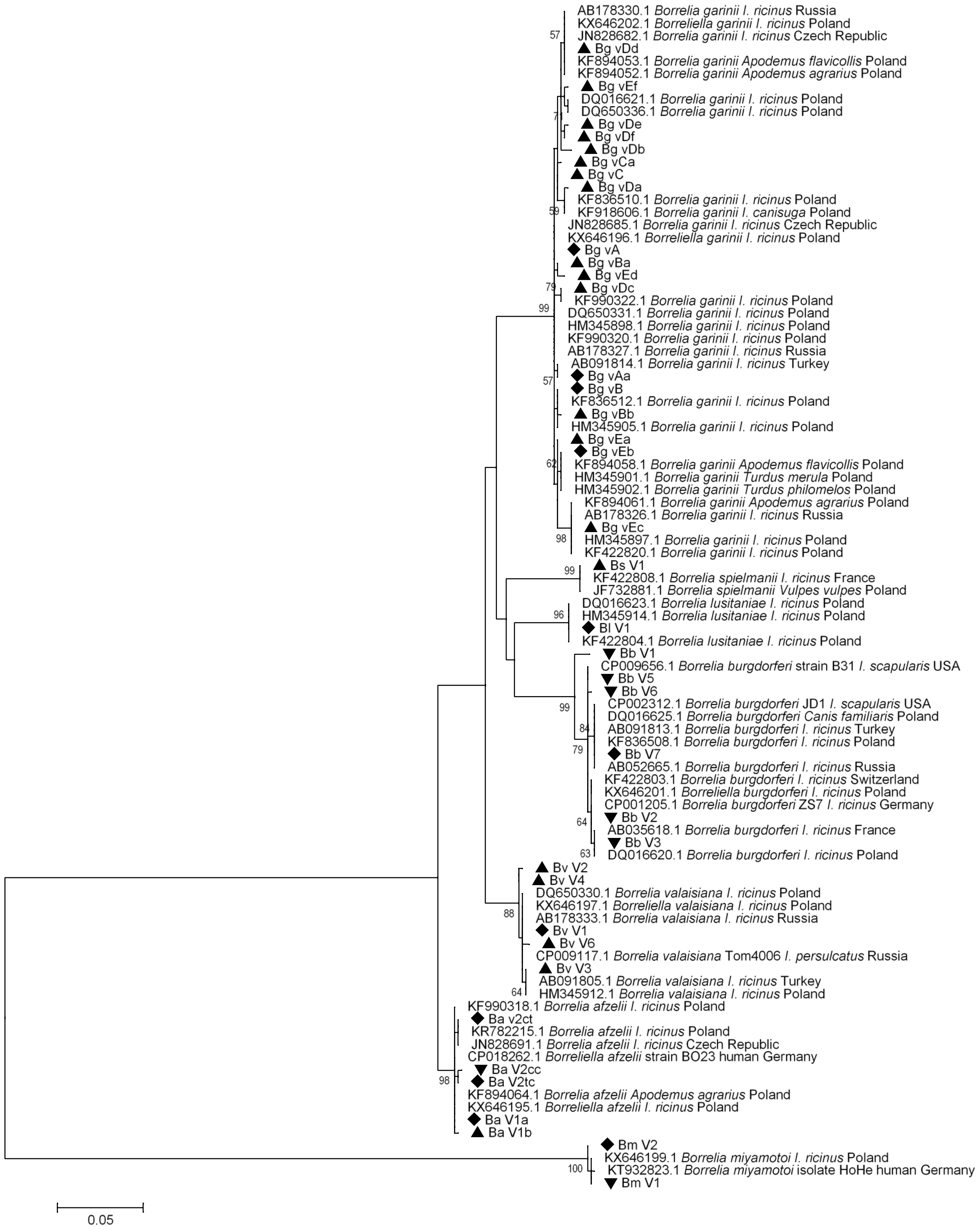
AB178330.1 Borrelia garinii I. ricinus Russia
KX646202.1 Borreliella garinii I. ricinus Poland
JN828682.1 Borrelia garinii I. ricinus Czech Republic
Bg vDd
KF894053.1 Borrelia garinii Apodemus flavicollis Poland
KF894052.1 Borrelia garinii Apodemus agrarius Poland
Bg vEf
DQ016621.1 Borrelia garinii I. ricinus Poland
DQ650336.1 Borrelia garinii I. ricinus Poland
Bg vDe
Bg vDf
Bg vDb
Bg vCa
Bg vC
Bg vDa
KF836510.1 Borrelia garinii I. ricinus Poland
KF918606.1 Borrelia garinii I. canisuga Poland
JN828685.1 Borrelia garinii I. ricinus Czech Republic
KX646196.1 Borreliella garinii I. ricinus Poland
Bg vA
Bg vBa
Bg vEd
Bg vDc
KF990322.1 Borrelia garinii I. ricinus Poland
DQ650331.1 Borrelia garinii I. ricinus Poland
HM345898.1 Borrelia garinii I. ricinus Poland
KF990320.1 Borrelia garinii I. ricinus Poland
AB178327.1 Borrelia garinii I. ricinus Russia
AB091814.1 Borrelia garinii I. ricinus Turkey
Bg vAa
Bg vB
KF836512.1 Borrelia garinii I. ricinus Poland
Bg vBb
HM345905.1 Borrelia garinii I. ricinus Poland
Bg vEa
Bg vEb
KF894058.1 Borrelia garinii Apodemus flavicollis Poland
HM345901.1 Borrelia garinii Turdus merula Poland
HM345902.1 Borrelia garinii Turdus philomelos Poland
KF894061.1 Borrelia garinii Apodemus agrarius Poland
AB178326.1 Borrelia garinii I. ricinus Russia
Bg vEc
HM345897.1 Borrelia garinii I. ricinus Poland
KF422820.1 Borrelia garinii I. ricinus Poland
Bs V1
KF422808.1 Borrelia spielmanii I. ricinus France
JF732881.1 Borrelia spielmanii Vulpes vulpes Poland
DQ016623.1 Borrelia lusitaniae I. ricinus Poland
HM345914.1 Borrelia lusitaniae I. ricinus Poland
Bl V1
KF422804.1 Borrelia lusitaniae I. ricinus Poland
Bb V1
CP009656.1 Borrelia burgdorferi strain B31 I. scapularis USA
Bb V5
Bb V6
CP002312.1 Borrelia burgdorferi JD1 I. scapularis USA
DQ016625.1 Borrelia burgdorferi Canis familiaris Poland
AB091813.1 Borrelia burgdorferi I. ricinus Turkey
KF836508.1 Borrelia burgdorferi I. ricinus Poland
Bb V7
AB052665.1 Borrelia burgdorferi I. ricinus Russia
KF422803.1 Borrelia burgdorferi I. ricinus Switzerland
KX646201.1 Borreliella burgdorferi I. ricinus Poland
CP001205.1 Borrelia burgdorferi ZS7 I. ricinus Germany
Bb V2
AB035618.1 Borrelia burgdorferi I. ricinus France
Bb V3
DQ016620.1 Borrelia burgdorferi I. ricinus Poland
Bv V2
Bv V4
DQ650330.1 Borrelia valaisiana I. ricinus Poland
KX646197.1 Borreliella valaisiana I. ricinus Poland
AB178333.1 Borrelia valaisiana I. ricinus Russia
Bv V1
Bv V6
CP009117.1 Borrelia valaisiana Tom4006 I. persulcatus Russia
Bv V3
AB091805.1 Borrelia valaisiana I. ricinus Turkey
HM345912.1 Borrelia valaisiana I. ricinus Poland
KF990318.1 Borrelia afzelii I. ricinus Poland
Ba v2ct
KR782215.1 Borrelia afzelii I. ricinus Poland
JN828691.1 Borrelia afzelii I. ricinus Czech Republic
CP018262.1 Borreliella afzelii strain BO23 human Germany
Ba V2cc
Ba V2tc
KF894064.1 Borrelia afzelii Apodemus agrarius Poland
KX646195.1 Borreliella afzelii I. ricinus Poland
Ba V1a
Ba V1b
Bm V2
KX646199.1 Borrelia miyamotoi I. ricinus Poland
KT932823.1 Borrelia miyamotoi isolate HoHe human Germany
Bm V1
57
59
79
99
57
62
98
99
96
99
84
79
64
63
88
64
98
100
0.05
