# Supplementary material for: Ticks and the city - are there any differences between city parks and natural forests in terms of tick abundance and prevalence of spirochaetes?
Source: Parasit Vectors. 2017 Nov 21;10:573. doi: 10.1186/s13071-017-2391-2 (PMC5697153; doi:10.1186/s13071-017-2391-2)
Supplement: Supplementary file 6 [file 13071_2017_2391_MOESM6_ESM.docx]

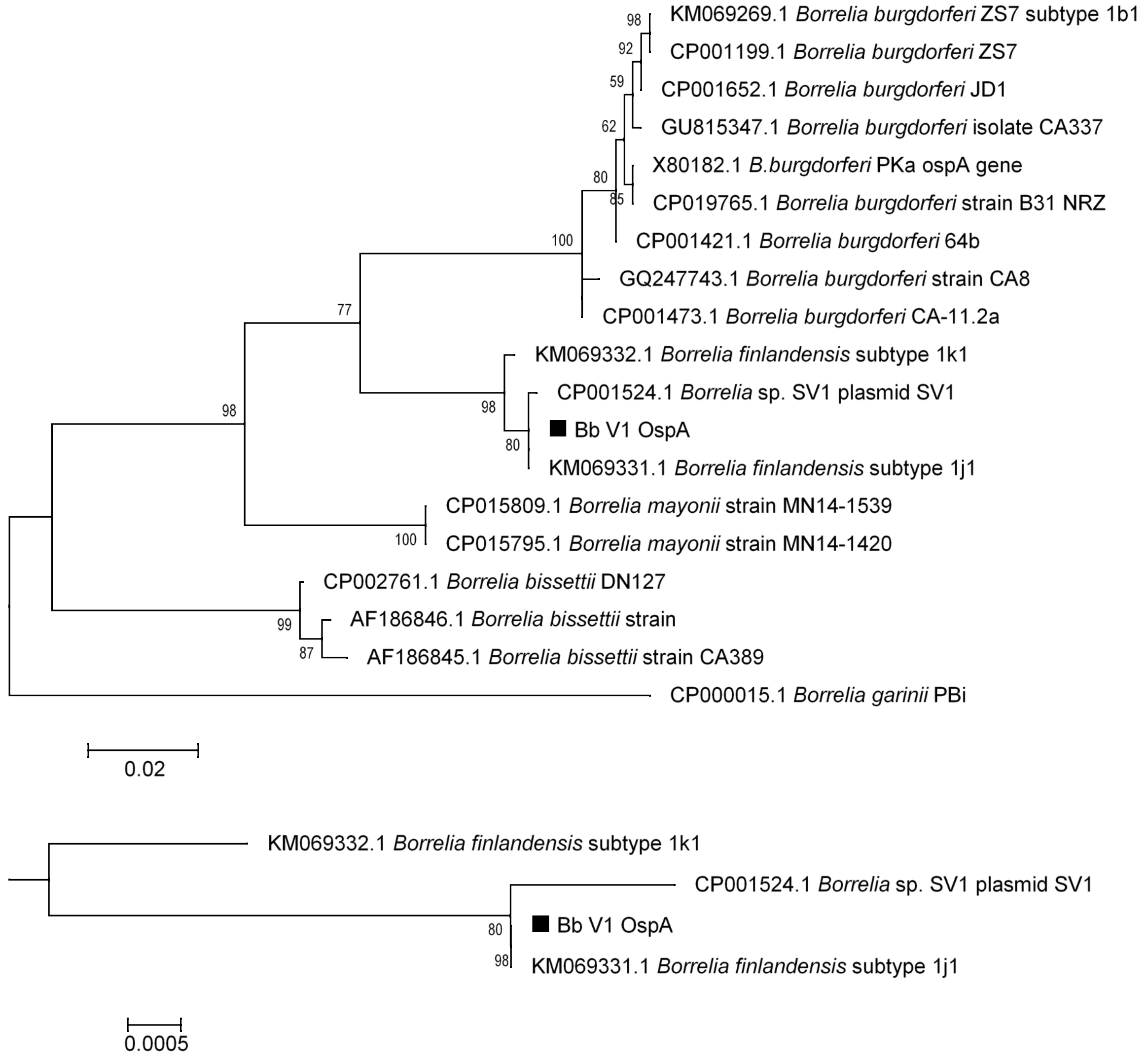

98
KM069269.1 Borrelia burgdorferi ZS7 subtype 1b1
92
CP001199.1 Borrelia burgdorferi ZS7
59
CP001652.1 Borrelia burgdorferi JD1
62
GU815347.1 Borrelia burgdorferi isolate CA337
80
X80182.1 B.burgdorferi PKa ospA gene
85
CP019765.1 Borrelia burgdorferi strain B31 NRZ
100
CP001421.1 Borrelia burgdorferi 64b
GQ247743.1 Borrelia burgdorferi strain CA8
77
CP001473.1 Borrelia burgdorferi CA-11.2a
KM069332.1 Borrelia finlandensis subtype 1k1
98
CP001524.1 Borrelia sp. SV1 plasmid SV1
98
Bb V1 OspA
80
KM069331.1 Borrelia finlandensis subtype 1j1
CP015809.1 Borrelia mayonii strain MN14-1539
100
CP015795.1 Borrelia mayonii strain MN14-1420
CP002761.1 Borrelia bissettii DN127
99
AF186846.1 Borrelia bissettii strain
87
AF186845.1 Borrelia bissettii strain CA389
CP000015.1 Borrelia garinii PBi
0.02
KM069332.1 Borrelia finlandensis subtype 1k1
CP001524.1 Borrelia sp. SV1 plasmid SV1
80
Bb V1 OspA
98
KM069331.1 Borrelia finlandensis subtype 1j1
0.0005
